# Dissecting the epigenomic dynamics of human fetal germ cell development at single-cell resolution

**DOI:** 10.1038/s41422-020-00401-9

**Published:** 2020-09-03

**Authors:** Li Li, Lin Li, Qingqing Li, Xixi Liu, Xinyi Ma, Jun Yong, Shuai Gao, Xinglong Wu, Yuan Wei, Xiaoye Wang, Wei Wang, Rong Li, Jie Yan, Xiaohui Zhu, Lu Wen, Jie Qiao, Liying Yan, Fuchou Tang

**Affiliations:** 1 Beijing Advanced Innovation Center for Genomics, Department of Obstetrics and Gynecology, Third Hospital, School of Life Sciences, Peking University, Beijing, 100871 China; 2 Biomedical Pioneering Innovation Center, Center for Reproductive Medicine, Ministry of Education, Key Laboratory of Cell Proliferation and Differentiation, Beijing, 100871 China; 3 Department of Pathophysiology, Guangdong Provincial Key Laboratory of Proteomics, School of Basic Medical Sciences, Southern Medical University, Guangzhou, Guangdong 510515 China; 4 Beijing Key Laboratory of Reproductive Endocrinology and Assisted Reproductive Technology, Beijing, 100191 China; 5 Key Laboratory of Assisted Reproduction, Ministry of Education, Beijing, 100191 China; 6 Peking-Tsinghua Center for Life Sciences, Peking University, Beijing, 100871 China; 7 Present Address: Stem Cell Program, Boston Children’s Hospital, Boston, MA 02115 USA; 8 Present Address: Department of Stem Cell and Regenerative Biology, Harvard University, Cambridge, MA 02138 USA

**Keywords:** Developmental biology, Epigenetics

## Abstract

Proper development of fetal germ cells (FGCs) is vital for the precise transmission of genetic and epigenetic information through generations. The transcriptional landscapes of human FGC development have been revealed; however, the epigenetic reprogramming process of FGCs remains elusive. Here, we profiled the genome-wide DNA methylation and chromatin accessibility of human FGCs at different phases as well as gonadal niche cells at single-cell resolution. First, we found that DNA methylation levels of FGCs changed in a temporal manner, whereas FGCs at different phases in the same embryo exhibited comparable DNA methylation levels and patterns. Second, we revealed the phase-specific chromatin accessibility signatures at the promoter regions of a large set of critical transcription factors and signaling pathway genes. We also identified potential distal regulatory elements including enhancers in FGCs. Third, compared with other hominid-specific retrotransposons, SVA_D might have a broad spectrum of binding capacity for transcription factors, including SOX15 and SOX17. Finally, using an in vitro culture system of human FGCs, we showed that the BMP signaling pathway promoted the cell proliferation of FGCs, and regulated the WNT signaling pathway by orchestrating the chromatin accessibility of its ligand genes. Our single-cell epigenomic atlas and functional assays provide valuable insights for understanding the strongly heterogeneous, unsynchronized, yet highly robust nature of human germ cell development.

## Introduction

Fetal germ cells (FGCs) are embryonic precursors of mature gametes, i.e. sperm or oocytes. Epigenomic reprogramming of FGCs is important for resetting the developmental potential, erasing epigenetic memory, and establishing unipotency in human germ cells.^1,2^ We and other groups have unveiled substantial global erasure of DNA methylation and unique chromatin state characteristics in human FGCs.^3–6^ However, all these studies were launched at developmental timescales and bulk levels. Recently, by interrogating the single-cell transcriptome of human FGCs and gonadal somatic cells, we found that human FGC development is asynchronous in later developmental stages.^7^ A female embryo after 18 weeks contains four phases of FGCs, i.e. mitotic, retinoid acid-responsive, meiotic prophase and oogenetic FGCs, and a male embryo after 9 weeks contains two phases of FGCs, i.e. mitotic, and mitotic-arrest FGCs. This indicates that it is essential to dissect the epigenome of FGC development in a phase-specific manner and at single-cell resolution. Besides, our previous study showed that IL13RA2 (also known as CD213α2) and PECAM1 (also known as CD31) were specific surface markers of meiotic prophase FGCs and oogenetic FGCs, respectively.^7^ This finding lays a foundation for enrichment of phase-specific FGCs by cell sorting. Thus, using scBS-seq and scCOOL-seq techniques,^8–11^ we systematically constructed genome-wide DNA methylation and chromatin accessibility maps for different phases of male and female FGCs at multiple critical developmental time points.

FGCs are capable of ‘self-renewal’ and differentiate into mature gametes.^12^ Gonadal somatic cells constitute the microenvironment of FGCs, i.e. the niche that has reciprocal interactions with FGCs to regulate the cell-fate commitment of germ cells. Our previous study has shown that BMP signaling pathway is activated in FGCs by ligands secreted from the gonadal somatic cells.^7^ However, the functional significance and epigenetic regulations of BMP signaling pathway involved in the FGC development remain largely unknown. To address these questions, we cultured 7-week male FGCs from testis in vitro with or without its antagonist LDN-193189 2HCl (LDN), and analyzed the transcriptional and epigenetic differences at single-cell level between these two conditions.

## Results

### Characteristics of DNA methylome reprogramming in human germ cells

With ethical approval, we obtained gonads from 14 human embryos and fetuses at 6–24 weeks post-fertilization to investigate the dynamic changes in the DNA methylome of FGCs as well as gonadal somatic cells (Supplementary information, Fig. S1a, b and Table S1). For gonads before 11 weeks, we collected C-KIT^+^ (CD117^+^) mitotic FGCs and C-KIT^−^ somatic cells using fluorescence-activated cell sorting (FACS).^13^ For testes after 17 weeks, we sorted C-KIT^high^ mitotic FGCs, C-KIT^low^ mitotically arrested FGCs and C-KIT^−^ gonadal somatic cells (Supplementary information, Fig. S1c). We collected IL13RA2^+^ meiotic prophase FGCs, PECAM1^+^ oogenetic FGCs, PECAM1^−^C-KIT^+^ mitotic FGCs and C-KIT^−^IL13RA2^−^PECAM1^−^ gonadal somatic cells from ovaries at 21 weeks (Supplementary information, Fig. S1d).^7^ We performed scBS-seq analysis of 461 sorted cells. 5.8 Gb sequencing data were generated for each individual cell and 2.7 Tb sequencing data were obtained in total. Then 413 single cells that passed the stringent quality control were retained for downstream analyses. On average, the bisulfite conversion rate was 99.6%, and 7.9 million CpG sites were covered in each individual cell, accounting for 14.1% of all CpG sites in the human genome (Supplementary information, Fig. S1e–h).

**Fig. 1:**
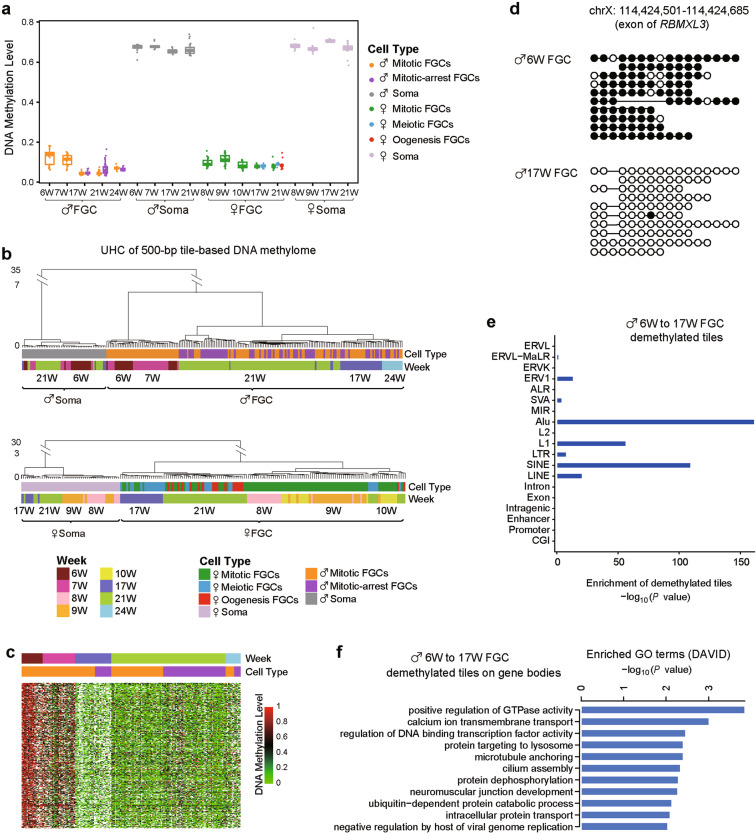
The DNA methylation dynamics of human FGCs. **a** Boxplot showing the dynamics of DNA methylation levels in the male and female FGCs and the somatic cells (Soma) from 6–24-week embryos. Each point represents the mean DNA methylation level of all the 1× CpG sites covered in a single cell. Cells of the same type at the same stage are plotted in a single box. **b** Unsupervised hierarchical clustering (UHC) analysis of 500-bp tile-based DNA methylomes of male (upper panel) and female (lower panel) gonadal cells. Only tiles covered in more than 70% of the cells are retained. The color bar on the first row represents the cell type and phases, and the color bar on the second row represents the gestational weeks. **c** Heat map showing the methylation levels on 1250 demethylated tiles identified from week 6 to week 17 during sequential phases of male FGC development. White boxes indicate data not detected. **d** Representative loci exhibiting substantial demethylation from week 6 to week 17 in male FGCs. Each row represents a read from the scBS-seq data, and each column represents a CpG site. The filled black dots represent the methylated CpG sites, whereas the open white dots represent the unmethylated CpG sites. **e** Analysis of the hypergeometric distribution of regions enriched for demethylated tiles from week 6 to week 17 in male FGCs. **f** DAVID (The Database for Annotation, Visualization and Integrated Discovery) GO (Gene Ontology) analysis of genes with demethylated tiles identified from week 6 to week 17 in the male FGCs located in the intragenic regions.

First, we analyzed the whole-genome levels of DNA methylation in FGCs at different phases and in gonadal somatic cells over developmental time (Fig. 1a). As previously reported, the genome of gonadal somatic cells was globally hypermethylated, and the mean methylation level was 67.4%.^3,4,6^ The mean methylation level of male FGCs decreased from 14.5% in 6-week testes to 4.9% in 17-week testes. Subsequently, the DNA methylation level gradually increased and reached 7.2% in 24-week testes, indicating that the global de novo DNA methylation process in male germ cells had been initiated. In 10–21-week ovaries, the DNA methylation level of the FGCs remained constant. Then, 208,861 500-bp genomic tiles in female FGCs and 128,773 tiles in male FGCs were used to perform unsupervised hierarchical clustering analysis and t-distributed stochastic neighbor embedding (t-SNE) analysis, respectively (Fig. 1b; Supplementary information, Fig. S2a, b). We found that FGCs were clearly separated from gonadal somatic cells and formed sub-clusters based mainly on their fetal ages. However, FGCs at the same fetal age but at different phases grouped together. Intriguingly, ovarian somatic cells were also divided into three clusters according to their fetal ages, suggesting that these cells might be granulosa cells at different developmental stages, i.e., early, mural and late granulosa cells.^7^ These results indicate that the DNA methylation dynamics of FGCs change mainly according to fetal age but do not differ significantly among different developmental phases of FGCs at the same fetal age.

We next explored the differentially methylated regions of FGCs between adjacent fetal ages (Supplementary information, Fig. S2c, d). The genomic tiles that exhibited dynamic changes in DNA methylation accounted for only a very small proportion compared with the proportion of tiles with stable DNA methylation levels. The number of methylation-increased or -decreased tiles between adjacent weeks was < 100, which was < 0.01% of the total tiles, except for the demethylated tiles in male FGCs from weeks 7–17. We further investigated the demethylated regions in male FGCs from weeks 6–17, namely, from the week with the highest DNA methylation to that with the lowest. We identified 1,250 demethylated tiles in the male FGC genome from weeks 6–17 (Fig. 1c, d). These tiles were primarily enriched in Alu and L1, the evolutionarily younger subfamily of SINEs and LINEs, respectively (Fig. 1e). Furthermore, we analyzed the genes with their gene body regions demethylated from weeks 6–17. These genes were strongly enriched in positive regulation of GTPase activity, calcium ion transmembrane transport, and regulation of DNA-binding transcription factor activity (Fig. 1f). The residual DNA methylation in germ cells could act as an epigenetic marker of transgenerational inheritance. We found that the 11,236 tiles with DNA methylation levels constantly ≥ 0.4 from weeks 6–24 in FGCs were substantially enriched at SVA, ERV1 and ERVK, implying that these transposable elements might be mediators of transgenerational epigenetic inheritance (Supplementary information, Fig. S2e, f). In addition, the residual DNA methylation was enriched at gene body regions of GTPase-regulation-related genes (Supplementary information, Fig. S2g). Highly ordered regulation of GTPase expression is crucial for proper cell growth and for preventing teratoma formation.^14^ We proposed that the residual methylation at gene body regions of GTPase-related genes guaranteed the proper expression of these genes during FGC development. However, we detected essentially no differences of DNA methylation between the different phases of FGCs in the same embryo.

DNA methylation of imprinting control regions (ICRs) modulates the transcription of imprinting genes in an allele-specific manner.^15^ Accurate erasure and re-establishment of imprinting plays a vital role in proper development of germ cells.^16^ We found that demethylation had occurred in a majority of ICRs in FGCs at as early as week 6 (Supplementary information, Fig. S3). Interestingly, the methylation level of ICRs for the *SNURF* gene gradually decreased in FGCs as fetal age increased. The residual methylation levels of the ICRs for *IGF2R, WDR27* and *PEG10* were higher than those of other ICRs in FGCs. In addition, their methylation levels were much higher in female FGCs than those in male ones. A similar pattern was also observed in mouse studies.^17^ In mouse FGCs, ICRs for *Peg10*, *Peg3*, and *Impact* were resistant to global DNA demethylation to some extent and exhibited a sex-differential pattern. These findings indicate that during FGC development, the DNA methylation dynamics of these ICRs are partially conserved between mouse and human.

Taken together, the DNA methylation dynamics of FGCs change mainly along fetal age. However, significant differences in DNA methylation were not observed among different phases (i.e. between mitotic phase and mitotic-arrest phase of male FGCs; and among mitotic phase, meiotic prophase and oogenetic phase of female FGCs) of gonadal FGCs, indicating that the sequential establishment of different phases FGCs in the same embryo may rely on epigenetic layers other than DNA methylation.

### Chromatin accessibility at promoter regions

**Fig. 2:**
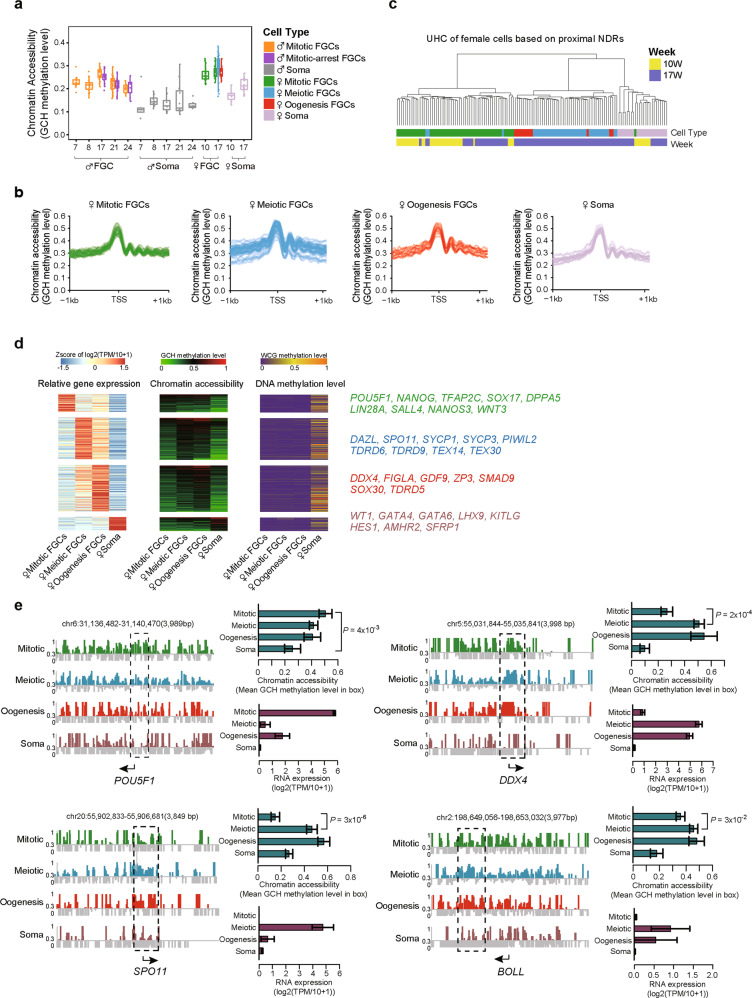
The chromatin accessibility profiles of proximal NDRs during female FGC development. **a** Boxplot showing the global chromatin accessibility of FGCs and somatic cells during sequential phases of development. **b** Average chromatin accessibility around the transcription start sites (TSSs) of all RefSeq genes among different cell types in female 17-week embryos. Each solid line represents a single cell. **c** Unsupervised hierarchical clustering analysis of female FGCs and somatic cells based on chromatin accessibility of 59,946 proximal NDRs detected among cell types or phases in female 10- and 17-week embryos. **d** Heat map showing the strong positive correlation between the expression of female developmental phase-specific genes and the openness of the chromatin states at proximal NDRs. Only regions with Pearson correlations higher than 0.6 are shown. A plot of the corresponding DNA methylation level of each promoter region is also shown. Master genes exhibiting a high correlation are listed in the right panel. The average methylation levels of each cell type in female 10- and 17-week embryos are used. The RNA-seq data are from our previous study.^7^ **e** Chromatin accessibility around the TSSs of *POU5F1*, *DDX4, SPO11* and *BOLL* in the female 17-week FGCs and the somatic cells are shown at a single-base resolution. The cytosines with their methylation level < 0.3 (but were detected in our single-cell samples) were shown in gray, and only the cytosines with their methylation level > 0.3 were shown in green/blue/red/brown based on the cell types. The representative differential open regions between cell types are highlighted in dashed rectangles. The mean chromatin accessibility of representative regions and RNA expression levels of the genes are shown in the right panel. The RNA-seq data of female 18-week embryos are from our previous study.^7^ The direction of the black arrow under each panel represents the direction of transcription. Data are shown as means ± SEM. Statistical significance analyses for the differences of GCH levels between two cell types were performed and the *P* values with Student’s *t-*test were shown here.

scCOOL-seq has been developed to simultaneously analyze chromatin accessibility and DNA methylation profiles from the same individual cell.^9^ To explore the epigenetic control of sequential phases of FGC development, 7–24-week FGCs and gonadal somatic cells were flow sorted as mentioned and the chromatin accessibility dynamics of 318 sorted cells were profiled using scCOOL-seq technique (Supplementary information, Figs. S1, S4a, b and Table S2). 6.7 Gb sequencing data were generated for each single cell and 2.1 Tb sequencing data were obtained in total. On average, 28.2 million GCH (GCA/GCT/GCC) sites and 3.5 million WCG (ACG/TCG) sites were covered in each individual cell, accounting for 12.2% of all GCH sites and 13.0% of all WCG sites in the human genome, respectively (Supplementary information, Fig. S4c–e). We calculated the global GCH level in each cell, and found that the chromatin of FGCs was much more accessible than that of gonadal somatic cells (Fig. 2a). Among female FGCs, meiotic prophase FGCs showed more heterogeneity in chromatin accessibility than mitotic FGCs and oogenetic FGCs, implying the highly dynamic features of chromatin states during meiosis (Fig. 2a, b). The global DNA methylation levels of gonadal cells assessed by scCOOL-seq and scBS-seq analyses were highly consistent (Supplementary information, Fig. S4f), further verifying the accuracy of our single-cell DNA methylome dataset.

**Fig. 3:**
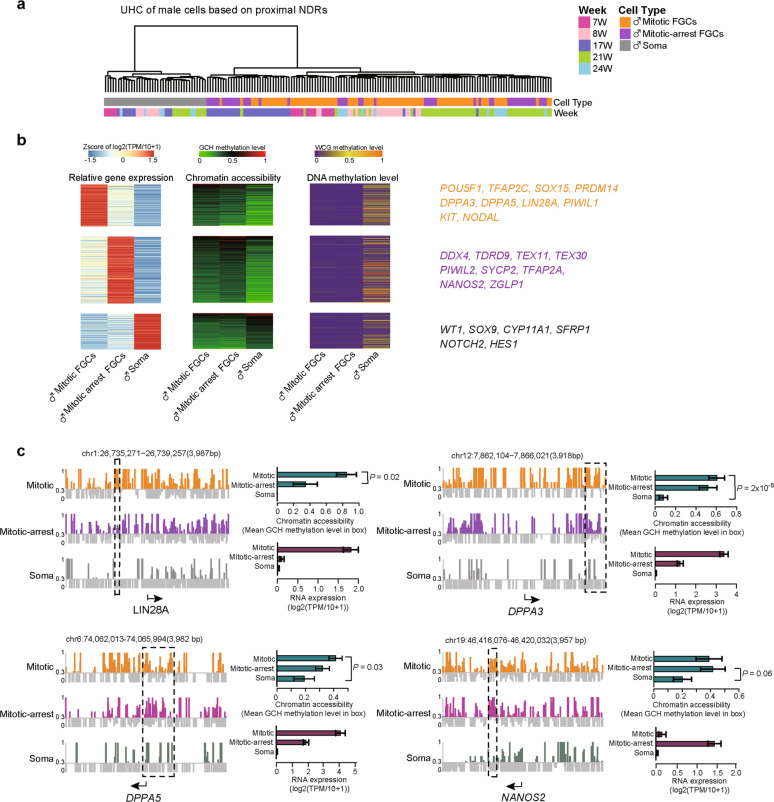
Chromatin accessibility features on proximal NDRs in male FGCs. **a** Unsupervised hierarchical clustering analysis of male FGCs and gonadal somatic cells based on the chromatin accessibility of 65,160 proximal NDRs detected among cell types or phases in male 7–24-week embryos. **b** Heat map showing the strong positive correlation between the expression of male developmental phase-specific genes and the openness of the chromatin states at proximal NDRs. Only regions with Pearson correlations higher than 0.6 are shown. A plot of the corresponding DNA methylation level of each promoter region is also shown. Master genes exhibiting high correlation are listed in the right panel. The average methylation levels of each cell type in male 7–24-week embryos were used. The RNA-seq data are from our previous study.^7^ **c** Representative plots of chromatin accessibility around the promoter regions in male 17-week FGCs and the somatic cells are shown at a single-base resolution. The cytosines with their methylation level < 0.3 (but were detected in our single-cell samples) were shown in gray, and only the cytosines with their methylation level > 0.3 were shown in orange/purple/gray-green based on the cell types. The representative differentially open regions between cell types are highlighted in dashed rectangles. The mean chromatin accessibility of representative regions and RNA expression levels of genes (estimated from male 21-week embryos in our previous study) are shown in the right panel. The direction of the black arrow under each panel represents the direction of transcription. Data are shown as means ± SEM. Statistical significance analyses for the differences of GCH levels between two cell types were performed and the *P* values with Student’s *t-*test were shown here.

Moreover, we conducted unsupervised hierarchical clustering analysis of human gonadal cells with 59,946 proximal nucleosome-depleted regions (NDRs). In general, gonadal cells were clustered together based on cell type and sequential development phases (Figs. 2c and 3a). Then, we identified 857 cell type-specific proximal NDRs in female FGCs and gonadal somatic cells and then conducted GO analysis of the corresponding genes (Supplementary information, Fig. S5a, b). The 265 female meiotic prophase FGC-specific proximal NDRs were enriched in NODAL signaling pathway genes. The 316 oogenetic FGC-specific proximal NDRs were enriched in stem cell differentiation, actin filament organization and responses to progesterone related genes. These results showed the highly phase-specific features of proximal NDRs and the potential regulation of transcriptome by these NDRs.

Thus, we subsequently screened the phase-specific genes whose expression patterns exhibited strong positive correlations with the chromatin accessibility at their promoter regions (Figs. 2d and 3b). The gene expression levels of a specific cell type at a similar phase were obtained from our previous study.^7^ Among these genes, the female mitotic FGC-specific genes included *DPPA5*, *LIN28A* and master transcription factors such as *POU5F1* (*OCT4*), *NANOG*, *TFAP2C* and *SOX17*. The meiotic prophase FGC-specific genes included *DAZL*, *SPO11*, *SYCP1*, *SYCP3*, *PIWIL2*, *TDRD9* and *TEX14*. *DDX4*, *FIGLA*, *GDF9*, *ZP3* and *TDRD5* were among the oogenetic FGC-specific genes. Female gonadal somatic cell-specific genes included *WT1*, *GATA4*, *GATA6*, *LHX9* and *AMHR2* (Fig. 2d, e; Supplementary information, Fig. S5c). Similarly, the genes with promoter regions specifically accessible in male mitotic FGCs included *OCT4*, *TFAP2C*, *SOX15*, *DPPA5* and *LIN28A*. The mitotically arrested FGC-specific genes included *DDX4*, *TDRD9*, *PIWIL2*, *TEX11* and *NANOS2* (Fig. 3b, c). For male gonadal somatic cells, the chromatin of the *SOX9* promoter region was specifically open, providing an epigenetic basis for the distinct expression of this gene in testes and for regulation of sex differentiation. Notably, several signaling pathway-related genes exhibited clear correlation between RNA expression and chromatin accessibility at their promoter regions of the corresponding genes. Our previous study^7^ has demonstrated that KIT signaling pathway is activated in FGCs by the ligand KITLG secreted from gonadal somatic cells. NOTCH signaling pathway is probably activated in somatic cells by ligands from FGCs. In addition, NODAL from mitotic FGCs might activate the NODAL signaling pathway in late-phase FGCs and probably controls the distribution of early- and late-phase FGCs in the peripheral and central regions of the same gonad, respectively.^7^ We found that the chromatin of the *KIT* promoter region was more open in mitotic FGCs than that in gonadal somatic cells, whereas the opposite trend was observed for the chromatin of the *KITLG* promoter (Figs. 2d and 3b). The chromatin of the *NOTCH2* and *HES1* promoters was more accessible in gonadal somatic cells than that in FGCs (Fig. 3b). In addition, the chromatin of the *NODAL* promoter was more accessible in mitotic FGCs (Fig. 3b). These results indicate that chromatin accessibility at promoter regions probably controls the specificity of master genes’ transcription and the direction of signaling regulation among different gonadal cell types. In summary, we identified the highly ordered promoter accessibility regulatory mechanisms of critical transcription factors and signaling pathway genes, which may contribute to proper developmental phase transition and maintenance of germ cell development.

### Distal regulatory elements in germ cell development

**Fig. 4:**
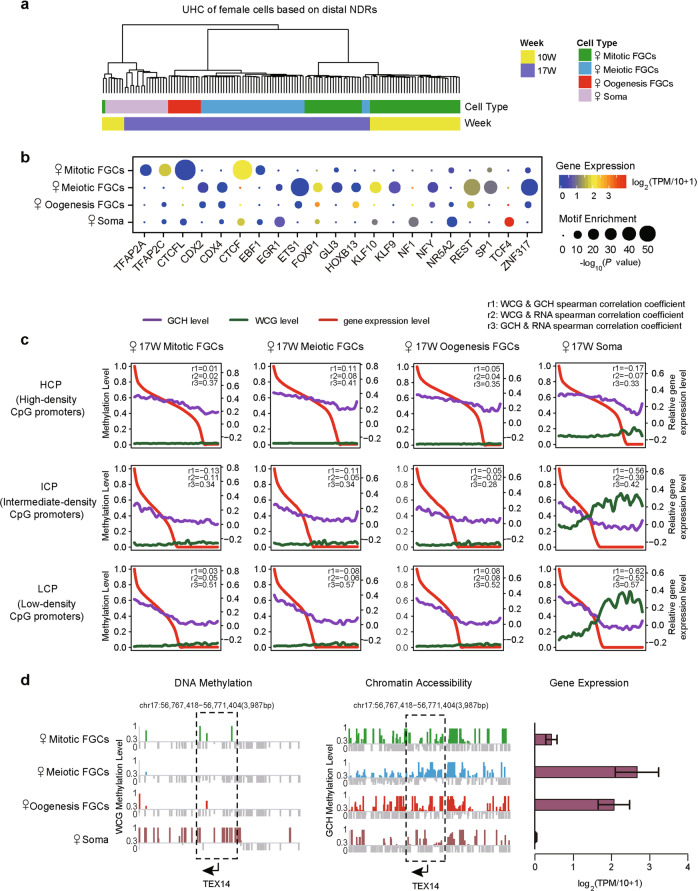
The distal regulatory network and the relationship among different omics during female FGC development. **a** Unsupervised hierarchical clustering analysis of female FGCs and somatic cells based on chromatin accessibility of 413,828 distal NDRs detected among cell types or phases in female 10- and 17-week embryos. **b** Motif analysis of cell type-specific open distal NDRs (see subsection titled ‘Differentially open regions’ in the ‘Materials and Methods’) in female 10- and 17-week embryos. Mitotic, meiotic prophase, and oogenetic FGCs and somatic cells are shown from top to bottom. Only the transcription factors with motif enrichment *P* values ≤ 10^−8^ in one cell type are shown. **c** The Spearman correlation among three omics at high-density CpG promoters (HCPs), intermediate-density CpG promoters (ICPs) and low-density CpG promoters (LCPs) in female 17-week embryos are shown. The average DNA methylation levels of the promoter regions were calculated based on WCG sites covered in a 1-kb region upstream of the TSS and a 0.5-kb region downstream of the TSS. The chromatin accessibility of the TSS regions were calculated based on GCH sites covered in a 200-bp region upstream of the TSS and a 100-bp region downstream of the TSS. The genes on the horizontal axis are ordered according to RNA expression level from left to right. The RNA-seq data of female 18-week embryos are from our previous study.^7^ **d** Representative plot of DNA methylation levels and chromatin accessibility around the *TEX14* promoter region are shown at single-base resolution. The cytosines with their methylation level < 0.3 (but were detected in our single-cell samples) were shown in gray, and only the cytosines with their methylation level ≥ 0.3 were shown in green/blue/red/brown based on the cell types. The representative differential regions between cell types are highlighted in dashed rectangles. The RNA-seq data of female 18-week embryos were used for estimation of gene expression levels.

**Fig. 5:**
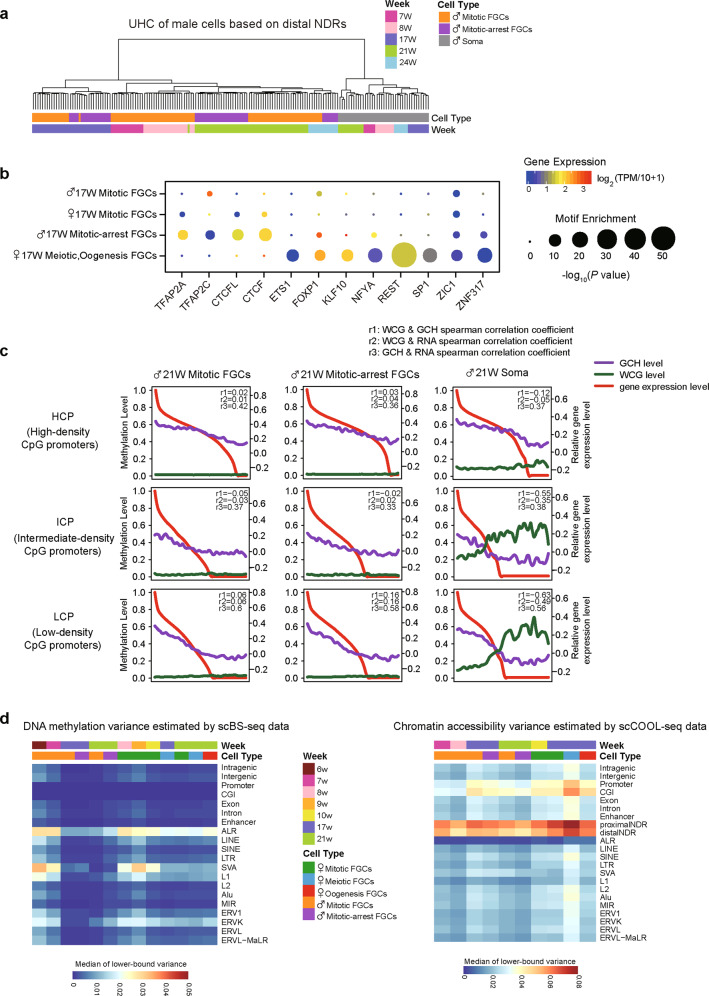
Chromatin accessibility features on distal NDRs and the relationship among three omics in male gonads. **a** Unsupervised hierarchical clustering analysis of male FGCs and somatic cells based on the chromatin accessibility of 703,315 distal NDRs detected among cell types or phases in male 7–24-week embryos. **b** Motif analysis of cell type-specific open distal NDRs (see the subsection titled ‘Differentially open regions’ in the ‘Materials and Methods’) between male mitotic FGCs and female mitotic FGCs, male mitotically arrested FGCs and female meiotic, oogenetic FGCs respectively in 17-week embryos. Only the transcription factors with motif enrichment *P* values ≤ 10^−5^ in one cell type are shown. **c** The Spearman correlation among three omics around the HCPs, ICPs and LCPs in male 21-week embryos are shown. The average DNA methylation levels (green lines) of the promoter regions were calculated based on WCG sites covered in the regions 1 kb upstream of the TSS and 0.5 kb downstream of the TSS. The chromatin accessibility (purple lines) of TSS regions are calculated based on GCH sites covered in the regions 200 bp upstream of the TSS and 100 bp downstream of the TSS. The genes on the horizontal axis are ordered according to RNA expression level (red lines) from left to right. The expression levels were estimated from the RNA-seq data of male 21-week embryos from our previous study.^7^ **d** The single-cell variance of DNA methylation levels at various functional genomic regions assessed using the scBS-seq data are shown in the left panel. The single-cell heterogeneity of chromatin accessibility at various genomic elements and repetitive elements using the scCOOL-seq data are shown in the right panel. See the subsection titled ‘Cell-to-cell variance among individual cells’ in the ‘Materials and Methods’ for details.

Next, we performed unsupervised hierarchical clustering analysis of female gonadal cells with 413,828 distal NDRs and male gonadal cells with 703,315 distal NDRs, respectively (Figs. 4a and 5a). We found that these NDRs clustered in a distinct phase-specific manner. Furthermore, we identified differential distal NDRs in female mitotic, meiotic, and oogenetic FGCs and gonadal somatic cells, respectively. Distal regulatory regions such as enhancers and insulators play critical roles in orchestrating precise spatiotemporal gene expression patterns during development. By combining the binding motif enrichment analysis with the RNA expression patterns of the corresponding transcription factors,^9^ we then depicted the distal regulatory network of human FGCs (Figs. 4b and 5b). Notably, the key primordial germ cell (PGC) transcription factor *TFAP2C* was specifically expressed and its binding motifs were clearly enriched in mitotic FGCs. *KLF10* and *FOXP1* were highly expressed and strongly enriched in meiotic prophase FGCs, whereas *HOXB13* was specifically expressed and enriched in oogenetic FGCs. As *KLF10* (*TIEG1*) is known to promote TGF-β signaling by downregulating negative feedback factors,^18,19^ we speculated that KLF10 binding to distal NDRs of the target genes was one possible mechanism for the enhancement of TGF-β signaling in female late-phase FGCs. FOXP1 and HOXB13 were known to participate in the androgen signaling pathway and promote oocyte maturation.^20–22^ In summary, we revealed the potential mechanisms of precise regulation of critical transcription factors and their cis-regulatory targets during human germ cell development.

### Relationships between DNA methylation, chromatin accessibility and gene expression

Promoters are categorized into high-density CpG promoters (HCPs), intermediate-density CpG promoters (ICPs) and low-density CpG promoters (LCPs). Generally, HCPs are associated with housekeeping genes, whereas LCPs are associated with tissue- and development-specific genes.^23,24^ First, we detected that HCPs had lower DNA methylation levels and higher chromatin accessibility than ICPs and LCPs (Supplementary information, Fig. S6). Next, we explored the relationships among these two epigenetic layers and gene expression patterns (Figs. 4c and 5c). In gonadal somatic cells, the negative correlation between the DNA methylation levels and RNA expression levels gradually increased from HCPs to ICPs and further to LCPs. The relationship in FGCs was not so prominent due to the relatively low levels of residual DNA methylation. However, both FGCs and somatic cells exhibited clear positive correlations between promoter chromatin accessibility and RNA expression, and the most significant correlations were observed at LCPs. In gonadal somatic cells, the negative correlation between DNA methylation and chromatin accessibility increased from HCPs to ICPs and further to LCPs. Although large-scale DNA demethylation renders the general decoupling of DNA methylation and RNA expression, we found several genes whose expression was perhaps orchestrated by both DNA methylation and chromatin accessibility, such as *TEX14*, which is specifically expressed in late-phase FGCs and important in gametogenesis (Fig. 4d). We detected DNA methylation around the transcription start site (TSS) of *TEX14* in mitotic FGCs, but not in late-phase FGCs. Correspondingly, the chromatin at TSS of *TEX14* had relatively high accessibility in meiotic and oogenetic FGCs, indicating that the synergetic effect of these two epigenetic layers controls the precise spatiotemporal expression of *TEX14* in germ cells.

### Variance in DNA methylation and chromatin accessibility among individual cells

Next we asked whether the DNA methylation reprogramming and chromatin remodeling processes are synchronized in individual FGCs at the same phase. We assessed the single-cell variance in DNA methylation levels and chromatin accessibility at various functional genomic regions (Fig. 5d).^11^ Early-stage (6–10 weeks) FGCs exhibited more variation in DNA methylation among individual cells than late-stage (17 and 21 weeks) FGCs, probably owing to the higher residual DNA methylation of these cells. We observed significant heterogeneity of DNA methylation on ALR, SVA, L1 and ERVK, indicating that the evolutionarily younger and more ‘dangerous’ transposable elements showed more variations of DNA demethylation and probably more epigenetic polymorphisms among different FGCs at the same phase in the same fetus. In addition, proximal NDRs, distal NDRs, promoters and CpG islands (CGIs) exhibited strong heterogeneity in chromatin accessibility, highlighting the highly dynamic features of the chromatin state of these genomic elements during germ cell development. Meiotic prophase FGCs had clearly stronger variations in chromatin accessibility at essentially all genomic elements than FGCs at other phases, emphasizing the highly divergent chromatin status of FGCs during meiosis.

### Epigenetic control of genomic elements and repeats

**Fig. 6:**
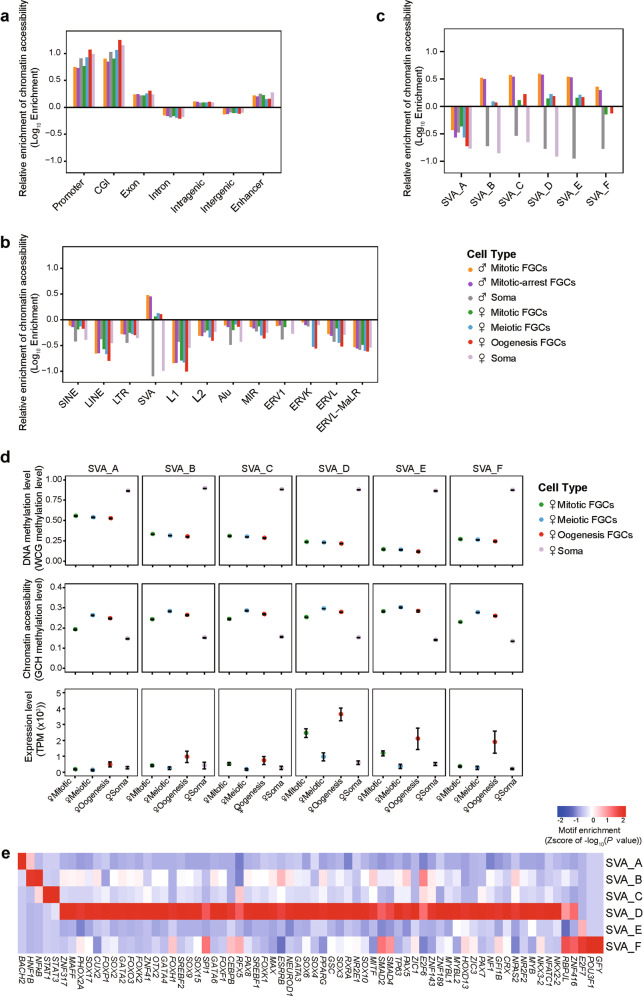
Open chromatin enrichment in different genomic elements. **a**, **b** Relative enrichment of open chromatin in each cell type in genomic regions (**a**) and repeats (**b**). **c** Relative enrichment of open chromatin in female 17-week embryos and male 21-week embryos in six SVA subfamilies. **d** Average DNA methylation levels and chromatin accessibility of six SVA subfamilies in female 17-week embryos. The expression levels were estimated from the RNA-seq data of female 18-week embryos from our previous study.^7^ **e** Heat map showing the relative enrichment of motifs located within the six SVA subfamilies. Only motifs with enrichment *P* values ≤ 10^−50^ in one subfamily are shown.

We then focused on the distinct features of these two epigenetic layers at different genomic elements including repetitive elements. We observed that CGIs exhibited significantly lower DNA methylation levels and higher chromatin accessibility than other genomic elements (Supplementary information, Fig. S6). Exons enriched more NDRs than introns, and intragenic regions enriched more NDRs than intergenic regions (Fig. 6a). Regarding repetitive elements, we found that ALR, SVA and ERVK continued to have distinctly higher DNA methylation levels than other repetitive elements in FGCs, likely leading to repression of their expression and laying a foundation for transgenerational inheritance of epigenetic information (Supplementary information, Fig. S7a).^25^

Notably, we found that SVAs enriched more NDRs compared with other repetitive elements. Besides, NDRs in SVAs were more enriched in FGCs rather than in gonadal somatic cells (Fig. 6b). Based on a phylogenetic analysis, SVAs are classified into six subfamilies (from SVA_A to SVA_F) according to their decreasing evolutionary ages.^26,27^ Compared with gonadal somatic cells, FGCs exhibited lower DNA methylation levels, higher chromatin accessibility and greater NDR enrichment at SVAs (Fig. 6c, d; Supplementary information, Fig. S7b). Apparently, SVA_A had higher DNA methylation levels and lower NDR enrichment than other SVA subfamilies, and consistent with this observation, SVA_A had lower RNA levels than other SVA subfamilies. We identified the genes containing SVA_A sequences at their gene body regions and found that they were associated with viral regulation, such as *CHMP3* and *RNF103-CHMP3*. We speculated that the higher residual DNA methylation levels of SVA_A perhaps control the expression of these viral regulation genes strictly, which is essential for genome integrity and stability. Moreover, SVA_D and SVA_E exhibited lower DNA methylation levels and higher RNA levels than other SVA subfamilies. SVA_D was highly expressed in mitotic FGCs and oogenetic FGCs. We further analyzed the motif enrichment of transcription factors in various SVA subfamilies and found that SVA-D might have a broad spectrum of binding capacity of transcription factors compared with other subfamilies. Among these transcription factors, SOX15 and SOX17 are specifically highly expressed in mitotic FGCs (Fig. 6e).^9,28^ Although human FGCs have evolved to retain higher residual DNA methylation in the evolutionarily older SVA_A to constrain the activity of this element, evolutionarily younger SVA_D and SVA_E continue to exhibit higher transcriptional activity, suggesting the existence of an ongoing host-invader arms race.^29,30^ Further studies are required to understand the sophisticated regulatory relationships between different SVA subfamilies and germ cell development.

### The function and epigenetic regulations of BMP signaling in FGCs

**Fig. 7:**
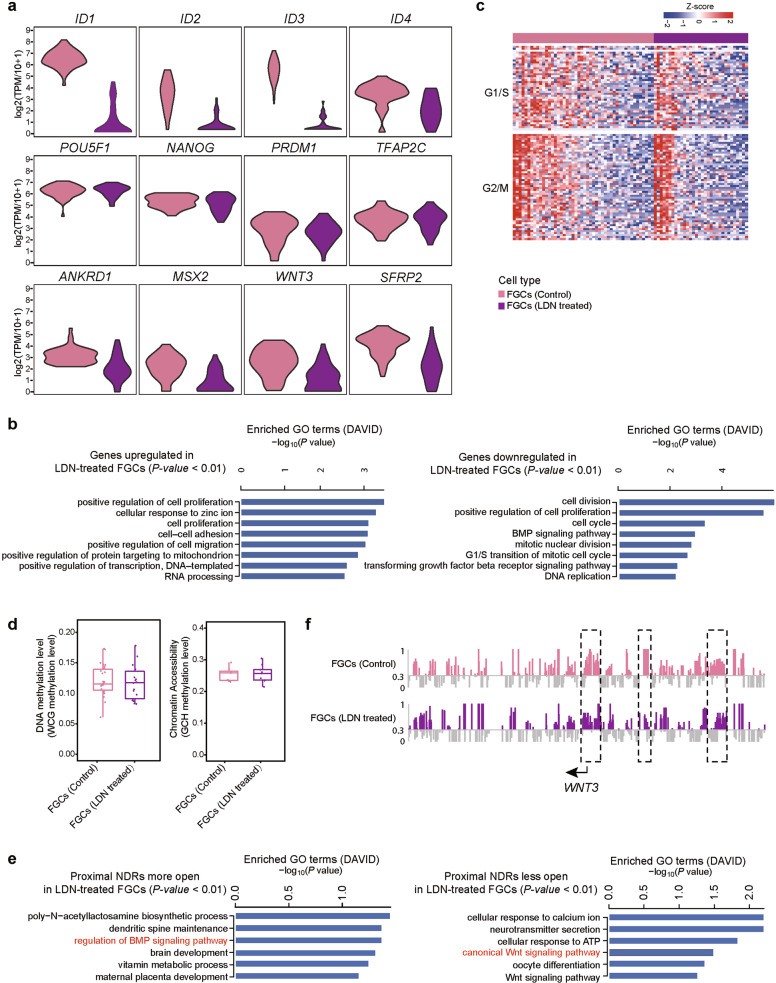
The transcriptional and epigenetic changes after the blockage of BMP signaling pathway. **a** Violin plots showing the expression level (log_2_(TPM/10 + 1)) of target genes of BMP signaling pathway, FGC master genes, apoptosis-related and WNT signaling-related genes in FGCs. Cells are colored by experimental conditions. **b** GO analysis of genes expressed higher (left panel) and lower (right panel) in LDN-treated FGCs compared with control FGCs. **c** Relative expression levels of cell-cycle related genes in control FGCs (left) and LDN-treated FGCs (right). **d** DNA methylation level and chromatin accessibility of control FGCs and LDN-treated FGCs. Each circle represents a single cell methylation level. **e** GO analysis of nearest genes to more open and less open proximal NDRs in LDN-treated FGCs compared with control FGCs. **f** Chromatin accessibility around the TSSs of *WNT3* in control FGCs and LDN-treated FGCs are shown at a single-base resolution. The representative differential open regions between cell types are highlighted in dashed rectangles.

Our previous study indicates that BMP signaling pathway might play an important role during FGC development.^7^ Here we utilized an in vitro culture system to explore the function and underlying epigenetic regulations of BMP signaling pathway in FGCs. We conducted optimized STRT-seq of in vitro cultured 7-week FGCs from testis with or without LDN,^7^ which is an antagonist of BMP signaling (Supplementary information, Tables S3 and S4). First, we analyzed the expression levels of BMP signaling-related genes in FGCs. We found that the level of BMP signaling target genes, *ID1-4* decreased significantly in LDN-treated FGCs compared with control FGCs (Fig. 7a), verifying the effective block of BMP signaling by LDN treatment. Next, LDN-treated FGCs expressed comparable *POU5F1*, *NANOG*, *PRDM1*, *TFAP2C* and other early FGC markers as control FGCs, implying that short-term blockage of BMP signaling pathway does not affect FGC cell’s identity (Fig. 7a). Moreover, we compared the specifically expressed genes between LDN-treated FGCs and control FGCs, and conducted GO analyses of them (Fig. 7b). We found that besides BMP signaling-related genes, the differentially expressed genes between them were enriched in cell proliferation regulations. Thus, we analyzed the expression pattern of cell cycle-related genes of FGCs in both conditions (Fig. 7c). Approximately half of the control FGCs were actively proliferating, which was consistent with FGCs in vivo, whereas only one quarter of FGCs treated with LDN were actively proliferating, implying that BMP signaling pathway promotes the cell proliferation of FGCs. In addition, it is reported that *ANKRD1* and *MSX2* are involved in regulation of cell apoptosis.^31^ We found that their expression levels were significantly lower in LDN-treated FGCs compared with those in control FGCs, implying that these two genes are potentially downstream targets of BMP signaling pathway (Fig. 7a). Collectively, we propose that the function of BMP signaling pathway in gonads might be balancing a reasonable pool of FGCs by coordinating their cell proliferation and apoptosis.

Furthermore, to study the BMP signaling’s potential epigenetic regulations of downstream target genes in FGCs, we performed scCOOL-seq of the cultured 7-week FGCs from testis with or without LDN (Supplementary information, Table S5). We found that the global DNA methylation levels of LDN-treated FGCs and control FGCs were comparable. There were no significant global differences between the chromatin accessibilities of LDN-treated FGCs and control FGCs (Fig. 7d). However, we detected significant differences in the distribution of proximal NDRs between these two conditions (Fig. 7e). Once the BMP signaling pathway was blocked, the chromatin accessibility at the promoters of WNT signaling-related genes, such as *WNT3* decreased in FGCs (Fig. 7f). Correspondingly, the expression level of *WNT3* was significantly lower in LDN-treated FGCs compared with that in control FGCs (Fig. 7a). These results imply that BMP signaling pathway might regulate WNT signaling pathway by orchestrating the chromatin accessibility and transcriptional expression of its ligand genes. In addition, we found that the expression level of *SFRP2* was significantly lower in LDN-treated FGCs compared with in control FGCs (Fig. 7a). *Sfrp2* was reported to activate Wnt signaling pathway and enhance cell proliferation in mouse intestine.^32^ Collectively, we propose that the existence of crosstalk between BMP and WNT signaling pathways during FGC development at both transcriptional and epigenetic levels helps to regulate the proper FGC development in vivo.

## Discussion

To our knowledge, this study is the first to precisely dissect the complex epigenomic regulatory networks of FGCs at sequential developmental phases and in the BMP-ablation condition. The progression of FGC development is determined by both the embryonic/fetal stages and FGC phases. From our study, the DNA methylation of FGCs primarily changed in a temporal manner during development. That is, different phases of FGCs at the same fetal stage have very similar DNA methylation levels and patterns whereas the same phases of FGCs at different fetal stages may have different levels of DNA methylation. However, the chromatin accessibility of FGCs was phase-specific, especially for critical transcription factors and signaling pathway genes at both promoter- and distal-regulatory regions. That is, the phase-specifically expressed genes in FGCs were mainly regulated by chromatin accessibility but not DNA methylation of their cis-regulated elements. However, we do find several potentially important genes whose expression might be orchestrated by these two epigenetic layers together. For example, during FGC phase transitions, the DNA methylation level of *TEX14* promoter decreased and meanwhile, its chromatin accessibility increased. Peter Hill et al. identified 45 genes in mouse FGCs, whose expression was gradually activated with the progression of DNA demethylation at their promoter regions, such as *Dazl*, *Sycp1-3* and *Mael.*^33^ Combined with these results in human and in mice, we propose that even though the transcriptional dynamics of FGCs is not coupled with DNA methylation reprogramming in global, a small set of critical genes are still tightly controlled by both DNA methylation and chromatin accessibility to guarantee the proper robust developmental process of FGCs.

SVAs are hominid-specific retrotransposons and dysregulation of SVAs could induce diseases in humans. For example, the SVA retrotransposition into *TAF1* locus leads to the reduced expression of *TAF1*. This aberrant transcription change is associated with X-linked Dystonia-Parkinsonism, a Mendelian neurodegenerative disease.^34^ To protect genomic integrity, the host has evolved various mechanisms to repress aberrant transcription of SVAs, such as using DNA methylation, piRNA pathway and lineage-specific KZNF genes.^6,35^ However, previous studies have detected the transcriptional expression of SVAs in early human embryos, oocytes and sperm.^36–38^ Our study showed that the chromatin of SVAs in FGCs was more accessible and their transcriptional level was higher compared with those in gonadal somatic cells. More interestingly, the expression level of SVA_D was the highest in mitotic FGCs, and the binding motifs of SOX15 and SOX17 were specifically enriched in SVA_D. These results imply that SVAs might act as a platform to recruit core transcription factors during FGC development, and potentially turn to be novel promoters and enhancers during the evolution process.^39^ The continuing arm race between the host and retrotransposons accelerates the generation of more complex regulations during development.^40,41^

The transition and maintenance of different phases of FGCs are tightly controlled by master transcription factor network and sophisticated cross-talks between different signaling pathways. Our work analyzed the proximal and distal chromatin accessibility characteristics of these critical players during FGC development in vivo. For example, we found that the chromatin accessibility at promoter regions of *POU5F1*, *NANOG*, *TFAP2C* and *SOX17* were specifically open in mitotic FGCs. And the binding motifs of *KLF10* and *FOXP1* were strongly enriched in the distal NDRs of meiotic prophase FGCs. Besides, using an in vitro culture system, we unveiled that BMP signaling pathway played a critical role in promoting cell proliferation and regulating cell apoptosis during FGC development. Although a study in Drosophila showed that Bmp signaling pathway is important for PGC to maintain its cell identity,^42^ we did not find significant differences in the expression levels of early FGC master genes between LDN-treated and control FGCs. However, we hypothesize that a long-term blockage of BMP signaling would induce FGC ablation owing to their aberrant cell proliferation. Furthermore, we found the potential crosstalk between BMP signaling pathway and WNT signaling pathway during human FGC development. Our work identified both transcriptional and epigenetic links between these two pathways and provided insightful clues for further investigations in this field.

In summary, our highly precise single-cell resolution epigenomic atlas of human FGC development sheds light on their largely-unexplored complex regulatory relationships among transcriptome, DNA methylome and chromatin accessibility, and paves the way for further effective germ cell culturing in vitro and for deciphering germ cell-related diseases, such as infertility and teratomas.^43,44^

## Materials and methods

### Collection of human fetal gonads

This study was approved by the Reproductive Study Ethics Committee of Peking University Third Hospital (2012SZ-013 and 2017SZ-043). All the human embryos between 6 and 24 weeks of gestation were voluntarily donated by donors who provided signed informed consent. We collected gonads from nine male embryos and five female embryos for scBS-seq analysis. Except that the 21-week male samples had three biological replicates, the 6- and 7-week samples had two biological replicates, all the remaining samples had one biological replicate. We collected gonads from six male embryos and two female embryos for scCOOL-seq analysis. Except that the 21-week male samples had two biological replicates, all the remaining samples had one biological replicate. In addition, 17-week, 21-week twins and 24-week male samples, as well as 10- and 21-week female samples were used for both scBS-seq and scCOOL-seq analyses.

### Isolation of FGCs at specific developmental phases using FACS

Fetal gonads were digested with an Accutase Cell Detachment Solution (Millipore, SCR005) at 37 °C for 5–15 min and the cell suspension was filtered through 70 μm Pre-Separation Filters (Miltenyi Biotec, 130-095-823; Supplementary information, Table S6). After centrifuging the cell suspension and discarding the supernatant, the cells were resuspended in L15 medium (plus 10% FBS). A PE-conjugated mouse anti-human C-KIT antibody (BD PharMingen, 555714) was used for fetal testis samples. A PE-conjugated mouse anti-human C-KIT (CD117) antibody (BD PharMingen, 555714), FITC-conjugated anti-human PECAM1 (CD31) antibody (Biolegend, 303104) and APC-conjugated anti-human IL13RA2 (CD213α2) antibody (Biolegend, 354406) were used for fetal ovary samples.^7^ Cell sorting was performed using a BD FACSAria instrument (Special Order Research Product), and the data were analyzed using FlowJo software (Tree Star).

### Culturing human gonadal FGCs

The 7-week fetal testes was digested with Accutase Cell Detachment Solution at 37 °C for 15 min, then resuspended in culture medium GMEM, supplemented with 15% v/v knock-out serum, 0.1 mM non-essential amino acid, 2 mM l-Glutamine, 0.1 mM β-Mercaptoethanol, 1 mM sodium pyruvate, 100 ng/mL LIF, 500 ng/mL BMP4, 100 ng/mL SCF, 50 ng/mL EGF and 10 μM Y27632.^44^ Gonadal cells were seeded into a 24-well plate (50,000 cells per well) and cultured at 37 °C in a humidified incubator with 5% CO2. After 6-h culturing, LDN-193189 2HCl was added with the final concentration 150 nM and cultured for 24 h. The control group was cultured for the same time. After enrichment of LDN-treated FGCs and control FGCs using C-KIT MACS antibody, we conducted STRT-seq and scCOOL-seq analysis of them.

### Single-cell PBAT library preparation and sequencing

Single cells were collected by mouth pipetting, transferred to lysis buffer, and lysed at 50 °C for 3 h. Genomic DNA was bisulfite converted and purified with EZ-96 DNA Methylation-Direct™MagPrep (Zymo, D5044). Bisulfite-converted DNA was amplified with biotinylated random primers and Klenow exo^−^ polymerase (Enzymatics, P7010-HC-L) for 7 rounds, and excess primers were removed by digestion with exonuclease I (NEB, M0293L). After second strand synthesis, the library was amplified with the KAPA HiFi HotStart ReadyMix (Kapa Biosystems, KK2602) for 13 cycles.^8^ The library was subjected to 150-bp paired-end sequencing on an Illumina HiSeq 4000 platform (sequenced by Novogene).

### Single-cell COOL-seq library preparation and sequencing

Single-cell COOL-seq was performed according to a published protocol.^9^ Briefly, single cells were collected with a mouth pipette and transferred to lysis buffer. The GpC methyltransferase M.CviPI and S-adenosylmethionine (NEB, M0227L) were added to the lysate and incubated at 37 °C for 45 min. After releasing the genomic DNA with protease (QIAGEN), EZ-96 DNA Methylation-Direct™MagPrep (Zymo, D5044) was used for bisulfite conversion. The subsequent procedures were the same as those described in the scBS-seq protocol.

### Quality control and alignment of raw reads

The same pipeline was used for the scBS-seq and scCOOL-seq data to perform the quality control and alignment of the raw reads. Briefly, raw reads were first trimmed of 9 bases of random primers, adapter sequences and low-quality bases using trim_galore (version 0.3.3). Then, the quality ensured reads were mapped to the human reference genome hg19 with Bismark (version 0.7.6) using our published pipeline, that is reads were aligned in a paired-end, non-directional module, then the unmapped reads were remapped in a single-end, non-directional module.^9^ Before methylation estimation and further analyses were performed, PCR duplicates were first removed with SAMtools (version 0.1.18).^45^

We got 461 scBS-seq libraries and 318 scCOOL-seq libraries and used strict quality control filters to ensure that we constituted a high-quality single cell profile. For scBS-seq, only libraries with reads mapping rate ≥ 20%, bisulfite conversion rate ≥ 99%, non-CpG methylation level ≤ 0.02 and with more than 2 million CpG sites covered in a single cell were retained. What’s more, for FGC libraries with DNA methylation level higher than 0.2 had unexpected DNA methylation patterns in the promoter regions of FGC specific markers, only single cell FGC libraries with DNA methylation level ≤ 0.2 were retained. For scCOOL-seq libraries, only libraries with reads mapping rate ≥ 20%, FGCs libraries with DNA methylation level ≤ 0.2 and with more than 1 million WCG sites and 10 million GCH sites covered in a single cell were retained. After filtering, we got 413 scBS-seq libraries and 302 scCOOL-seq libraries for further analysis.

### Estimation of methylation levels

For scBS-seq data, the DNA methylation level of each covered cytosine on both strands was estimated by calculating the proportion of C divided by the depth of the same site (the number of methylated and unmethylated reads). For scCOOL-seq data, we employed the pipeline published by our group and other laboratories.^5,9,46,47^ That is, we used WCG (ACG/TCG) to estimate the DNA methylation level and GCH (GCA/GCC/GCT) to quantify the chromatin accessibility. We used 1× depth data to perform all the single-cell analyses. The methylation level of a region or a single cell was determined as the mean methylation level of all specific types of C sites covered in a region or in a single cell. We also used the tile-based method to bin the genome into contiguous windows to compare the methylation levels among single cells. For scBS-seq data, we used 500-bp windows with a coverage of at least 3 CpG sites. Pearson’s correlation analysis was performed using the R ‘cor’ function. The unsupervised hierarchical clustering analysis was conducted with the R ‘hclust’ function using the ‘ward’ method, whereas the tile-based or NDR-based t-SNE analysis was conducted using the R ‘tsne’ function. For the tile-based analysis of the DNA methylation levels in the scBS-seq data, we used regions covered in more than 70% of cells to perform the t-SNE analysis and unsupervised hierarchical clustering analysis. On the other hand, to determine chromatin accessibility at proximal and distal NDRs, we used all NDRs to conduct the unsupervised hierarchical clustering analysis. Genomic region annotations and repetitive elements used in this study were downloaded from the UCSC genome browser as described in a previous study.^4^ The annotations for the human enhancer regions were obtained from a previous study.^48^ The imprinted differentially methylated region (DMR) coordinates were also downloaded.^15^

### Differentially methylated regions

For the scBS-seq data, we compared the average DNA methylation levels of 500-bp tiles covered in two continuous phases or cell types. If the mean methylation level of the former was > 0.75 and that of the latter was < 0.25 with a *P* value ≤ 0.05 (the *P* value was adjusted for multiple comparisons using the ‘fdr’ method), then this site was termed a demethylated tile. If the mean methylation level of the former was < 0.25 and that of the latter was > 0.75 with a *P* value ≤ 0.05, then this site was termed a de novo methylation tile. The remaining sites were termed stable ones. Only tiles covered in more than 10% of cells in both stages or cell types were used for comparison.

### Detection of NDRs

We merged single cells from the same phase or type to accurately define their specific open chromatin regions and then analyzed the chromatin states of those regions in each individual cell. A sliding window chi-square test based on the GCH methylation level described in a previous study was used to detect NDRs. Briefly, the number of reads supporting C and T at each GCH site was counted in each 100-bp window with a spacing of 20-bp steps. Only windows with a significantly higher GCH methylation level (*P* value ≤ 10^−20^) compared to the whole-genome background and longer than 140 bp with at least five GCH sites covered were defined as a NDR. The NDRs were classified into 2 groups based on the distance between their center and the transcription start site (TSS). Proximal NDRs were located within 2 kb upstream and 2 kb downstream of the TSS, whereas distal DNRs were located at least 2 kb away from the TSS.

### Differentially open regions

When we compared the differences in openness between two cell types, only the regions that were covered in more than 40% of the cells in both groups, that exhibited a difference > 0.2 in the GCH methylation level, and for which more than half of the covered cells were open in only one group were termed as differentially open regions (the GCH methylation levels of relatively closed regions were ≤ 0.3, whereas the GCH methylation levels of relatively open regions were ≥ 0.5).

### Cell type-specific genes associated with open chromatin

The transcriptome data were obtained from our previous study.^7^ For both transcriptome and epigenome data, we aggregated single cells from the same cell type and the same/adjacent developmental age as a bulk sample. Then we aligned them and conducted correlation analysis. We calculated the Pearson correlation coefficients between gene expression level and the chromatin accessibility of the proximal NDRs to identify cell type-specific genes associated with highly open chromatin states. Only genes with Pearson correlation coefficients > 0.6 were selected and are shown in the heat map.

### Motif analysis

‘findMotifsGenome.pl’ in HOMER (version 4.9.1) was used to search for TFs that were potentially bound to open chromatin regions of each cell phase or type or SVA subfamilies with the command ‘-size 2000 -len 8 -S 100’.^49^

### Cell-to-cell variance among individual cells

The variance in DNA methylation levels in the scBS-seq data were calculated using a 3000-bp sliding window with a 600-bp step, as described in a previously reported method, with minor revisions.^11^ That is, only windows with coverage of more than 3 CpG sites and covered in more than 30% of the cells were analyzed. The lower variance bound of the chi-squared confidence interval of the variance estimator with a confidence level of 0.95 was used to estimate variance among single cells in each region. Meanwhile, the chromatin accessibility variance in scCOOL-seq data was estimated as previously reported.^50^ That is, a 200-bp window with a 100-bp step was used to calculate the chromatin accessibility variance. Only windows with more than 5 GCH sites covered were used. When < 10 FGCs were available at a specific phase in a certain week, these cells were excluded from this analysis.

## Supplementary information

Supplementary information, Fig. S1

Supplementary information, Fig. S2

Supplementary information, Fig. S3

Supplementary information, Fig. S4

Supplementary information, Fig. S5

Supplementary information, Fig. S6

Supplementary information, Fig. S7

Supplementary information, Table S1

Supplementary information, Table S2

Supplementary information, Table S3

Supplementary information, Table S4

Supplementary information, Table S5

Supplementary information, Table S6

## Data Availability

All the raw sequencing data have been deposited in GSA for human under accession number HRA000186, while the processed data were deposited in the GEO database under accession number GSE107714.
