# Supplementary material for: Dissecting the epigenomic dynamics of human fetal germ cell development at single-cell resolution
Source: Cell Res. 2020 Sep 3;31(4):463–77. doi: 10.1038/s41422-020-00401-9 (PMC8115345; doi:10.1038/s41422-020-00401-9)
Supplement: Supplementary file 1 — Supplementary information, Fig. S1 [file 41422_2020_401_MOESM1_ESM.pdf]

a

Gender	Male embryos								
Week	6 W		7W		17W	21W			24W
	e1	e2	e1	e2		e3	twin1	twin2	
Mitotic FGCs	10	7	21	5	16	13	15	14	7
Mitotic arrest FGCs					13	16	17	17	5
Soma	14	1	9	0	4	8	8	6	0
Sum	24	8	30	5	33	37	40	37	12
Total	226								

b

Gender	Female embryos				
Week	8W	9W	10W	17W	21W
Mitotic FGCs	20	28	20	7	17
Meiotic FGCs				14	15
Oogenesis FGCs				0	18
Soma	13	15	0	6	14
Sum	33	43	20	27	64
Total	187				

c

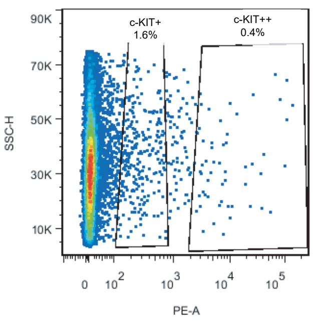

d

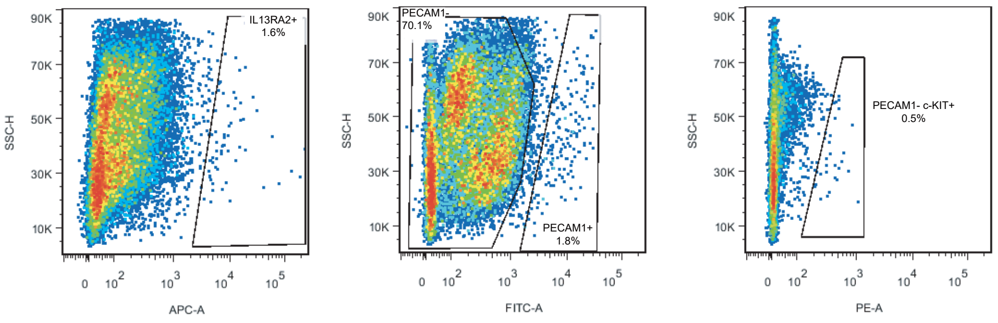

e

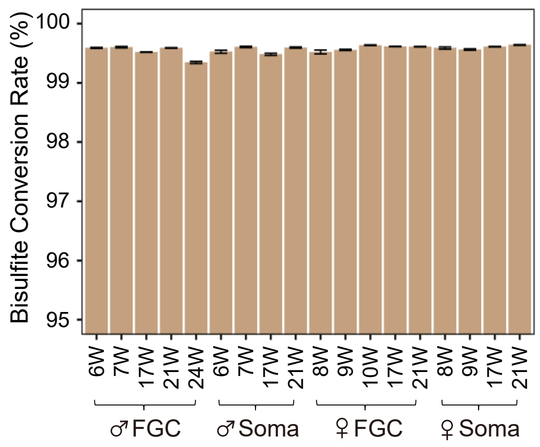

f

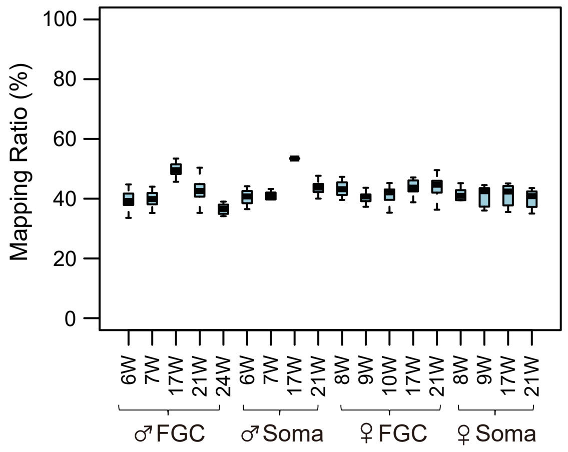

g

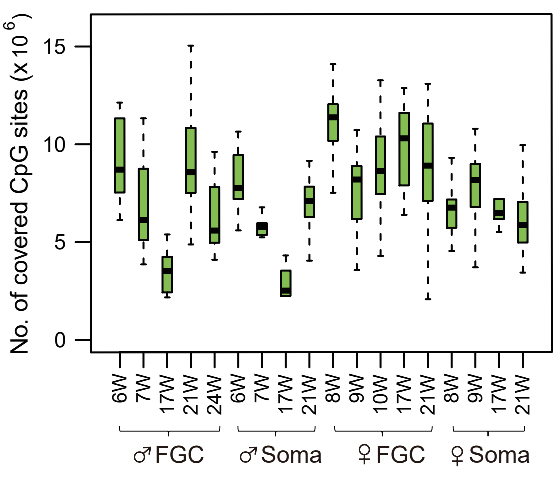

h

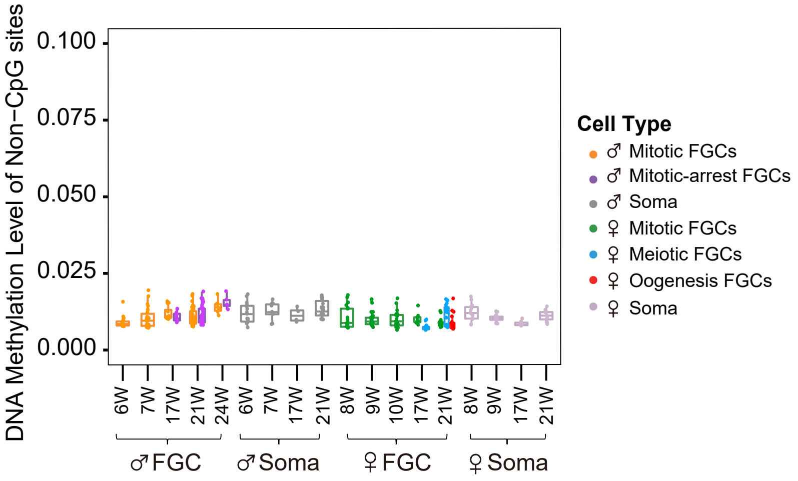

Fig. S1: Quality control and sequencing statistics of scBS-seq data.

a and b Overview of the numbers of each type of human male **(a)** and female **(b)** FGCs and somatic cells analyzed using scBS-seq.

c Cell sorting plot of the male mitotic FGCs (C-KIT^{high}) and the mitotically arrested FGCs (C-KIT^{low}).

d Cell sorting plot of the female meiotic prophase FGCs (IL13RA2^{+}), oogenetic FGCs (PECAM1^{+}) and mitotic FGCs (PECAM1^{-} C-KIT^{+}).

e Histograms showing the bisulfite conversion rate of the scBS-seq data. Data are shown as the mean ± s.e.m.

f Boxplot showing the read mapping ratio of the scBS-seq data.

g Boxplot of the number of CpG sites covered in each single cell during development at 1× sequencing depth.

h The global dynamics of the non-CpG DNA methylation levels in different cell types over time.
