# Supplementary material for: Dissecting the epigenomic dynamics of human fetal germ cell development at single-cell resolution
Source: Cell Res. 2020 Sep 3;31(4):463–77. doi: 10.1038/s41422-020-00401-9 (PMC8115345; doi:10.1038/s41422-020-00401-9)
Supplement: Supplementary file 2 — Supplementary information, Fig. S2 [file 41422_2020_401_MOESM2_ESM.pdf]

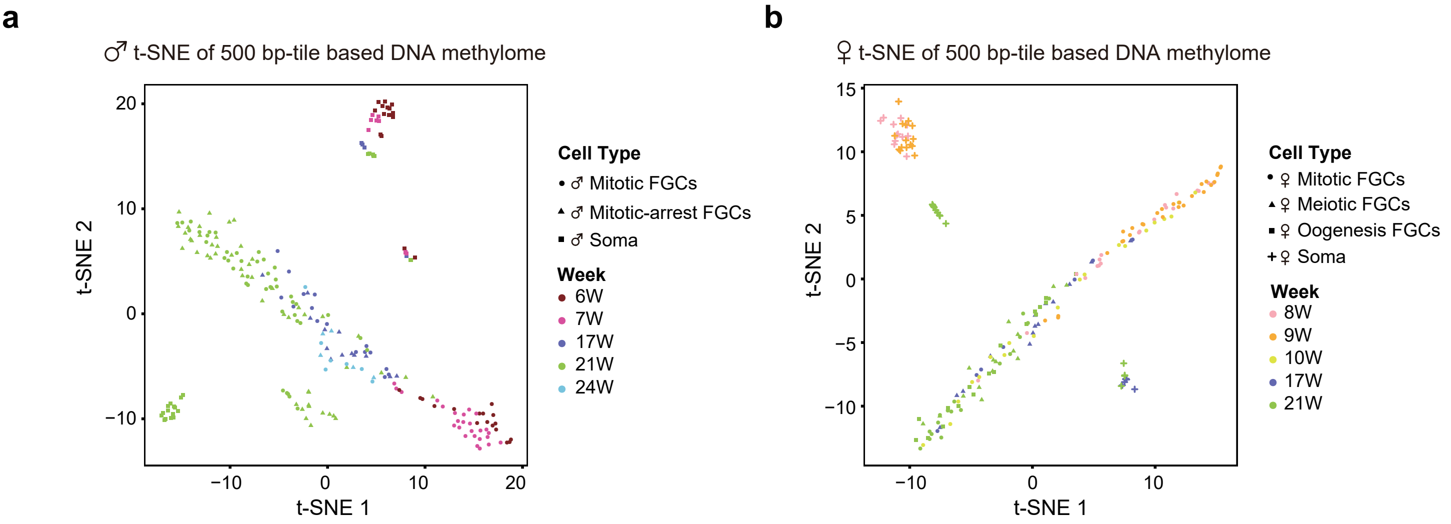

c

	♂ 6W .vs.7W	♂ 7W .vs.17W	♂ 17W .vs.21W	♂ 21W .vs.24W	♀ 8W .vs.9W	♀ 9W .vs.10W	♀ 10W .vs.17W	♀ 17W .vs.21W
Stable Tiles	2,156,087	1,261,899	1,307,460	1,712,996	2,429,676	2,354,568	2,422,198	2,426,523
Increased Tiles	26	22	3	17	50	23	30	12
Decreased Tiles	78	467	38	7	15	85	47	20

d

	♂ 17W mitotic vs. mitotic arrest	♂ 21W mitotic vs. mitotic arrest	♀ 21W mitotic vs. meiotic	♀ 21W meiotic vs. oogenesis	♀ 21W mitotic vs. oogenesis
Stable Tiles	734,569	2,407,891	2,265,292	2,466,758	2,469,286
Increased Tiles	56	2	47	6	9
Decreased Tiles	17	NA	14	41	7

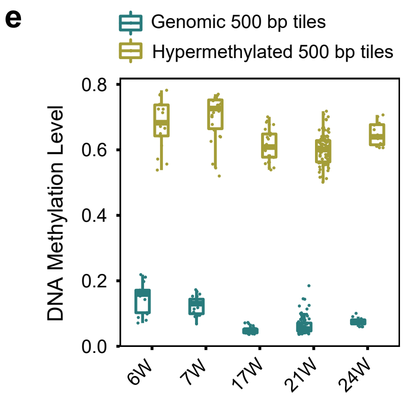

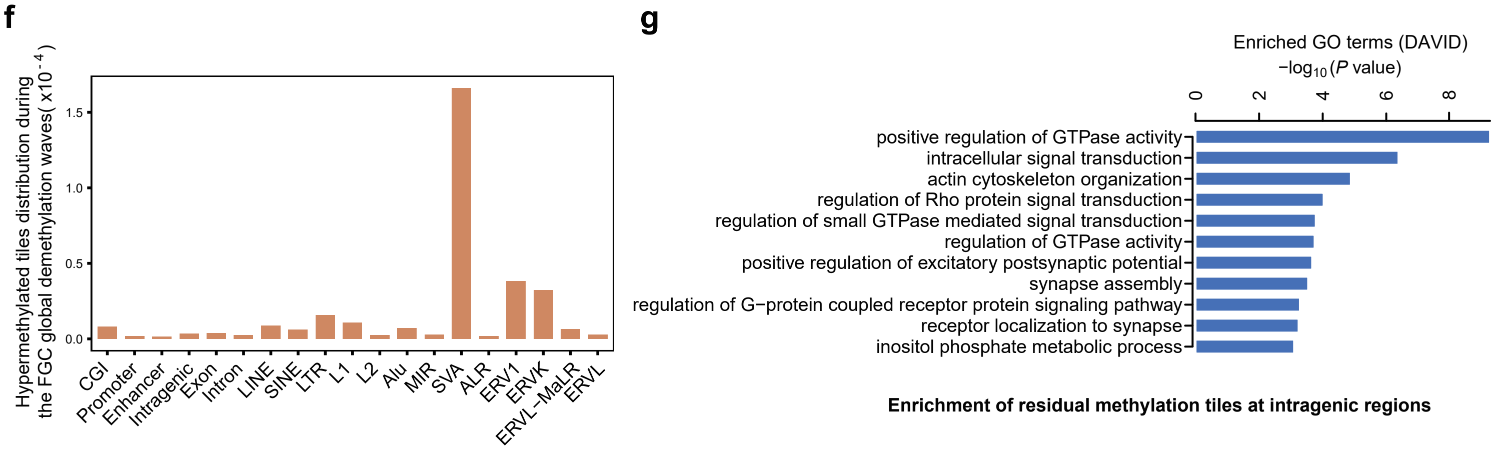

Fig. S2: The DNA methylation dynamics and tiles that remained hypermethylated during the demethylation waves of FGC development.

a and b t-SNE plots of the 500-bp tile-based DNA methylomes of the male **(a)** and female **(b)** FGCs and the somatic cells from 6- to 24-week embryos. These analyses are based on 128,773 tiles covered in more than 70% of all the male cells and 208,861 tiles covered in more than 70% of all the female cells.

c and d The number of tiles with stable, increased and decreased methylation levels during sequential gestational weeks of development **(c)**, and among different phases of FGCs within the same stage **(d)**. When less than 10 FGCs were available at a specific phase in a certain week, these cells were excluded from this analysis.

e Boxplot showing the methylation levels of the 11,236 residual tiles in FGCs with methylation levels that were constantly ≥ 0.4 between week 6 and week 24 compared with those of the global tiles. Only tiles covered in more than 20% of the cells in each stage were retained.

f Region distribution of 11,236 tiles that remained hypermethylated during the FGC global demethylation waves.

g GO analysis of genes with the residually methylated tiles located within the intragenic regions.
