# Supplementary material for: Dissecting the epigenomic dynamics of human fetal germ cell development at single-cell resolution
Source: Cell Res. 2020 Sep 3;31(4):463–77. doi: 10.1038/s41422-020-00401-9 (PMC8115345; doi:10.1038/s41422-020-00401-9)
Supplement: Supplementary file 3 — Supplementary information, Fig. S3 [file 41422_2020_401_MOESM3_ESM.pdf]

a

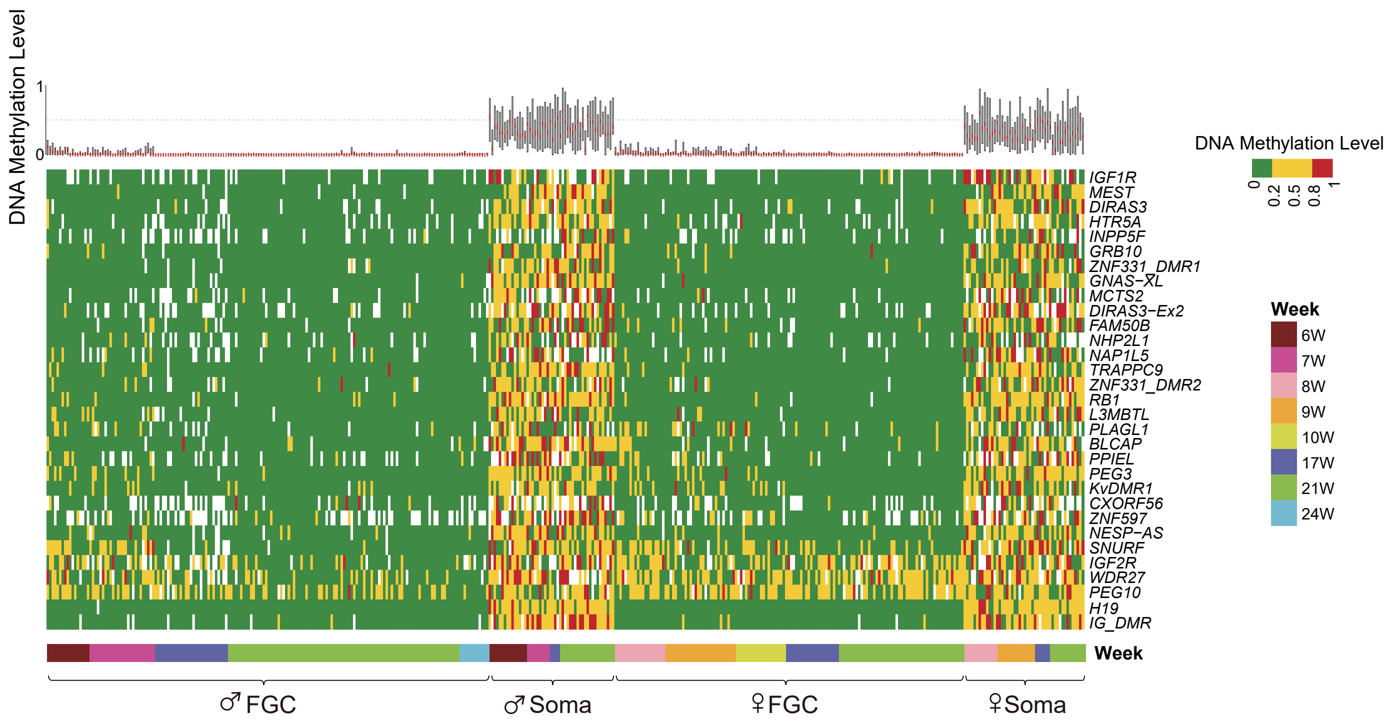
DNA Methylation Level
1
0
IGF1R
MEST
DIRAS3
HTR5A
INPP5F
GRB10
ZNF331_DMR1
GNAS−XL
MCTS2
DIRAS3−Ex2
FAM50B
NHP2L1
NAP1L5
TRAPPC9
ZNF331_DMR2
RB1
L3MBTL
PLAGL1
BLCAP
PPIEL
PEG3
KvDMR1
CXORF56
ZNF597
NESP−AS
SNURF
IGF2R
WDR27
PEG10
H19
IG_DMR
Week
♂FGC
♂Soma
♀FGC
♀Soma
DNA Methylation Level
0
0.2
0.5
0.8
1
Week
6W
7W
8W
9W
10W
17W
21W
24W

b

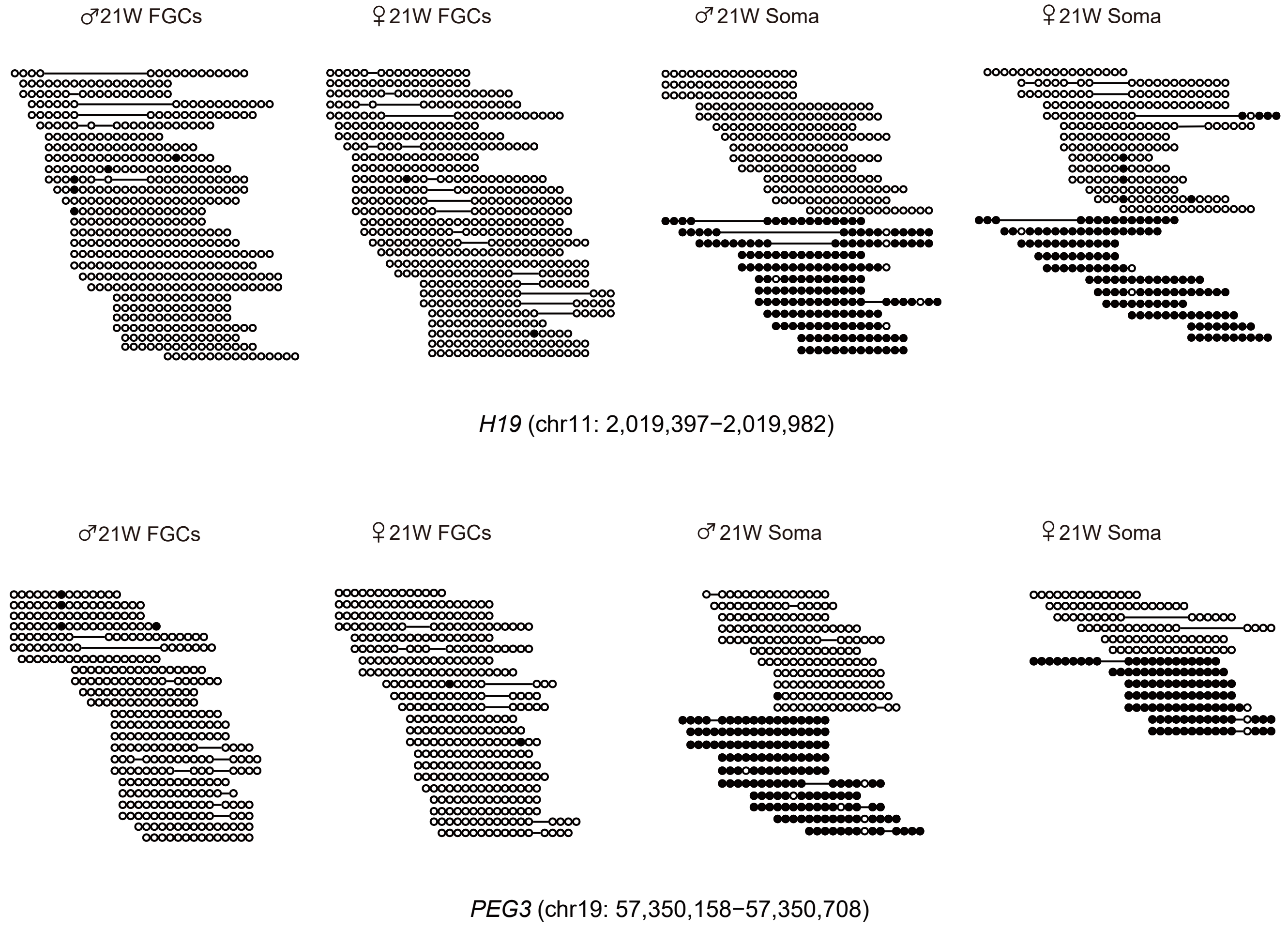
♂21W FGCs
♀21W FGCs
♂21W Soma
♀21W Soma
H19 (chr11: 2,019,397−2,019,982)
♂21W FGCs
♀21W FGCs
♂21W Soma
♀21W Soma
PEG3 (chr19: 57,350,158−57,350,708)

Fig. S3: DNA methylation levels of known ICRs.

a Heat map showing the DNA methylation levels of known ICRs in FGCs and somatic cells over time.

b Representative DNA methylation levels at the loci for the paternally imprinted gene *H19* and maternally imprinted gene *PEG3* in 21-week male and female FGCs and somatic cells. Each row represents a read from the scBS-seq data, and each column represents a CpG site. The filled black dots represent the methylated CpG sites, whereas the open white dots represent the unmethylated CpG sites.
