# Supplementary material for: Dissecting the epigenomic dynamics of human fetal germ cell development at single-cell resolution
Source: Cell Res. 2020 Sep 3;31(4):463–77. doi: 10.1038/s41422-020-00401-9 (PMC8115345; doi:10.1038/s41422-020-00401-9)
Supplement: Supplementary file 4 — Supplementary information, Fig. S4 [file 41422_2020_401_MOESM4_ESM.pdf]

a

Gender	Male embryos					
Week	7W	8W	17W	21W		24W
				twin1	twin2	
Mitotic FGCs	14	21	17	19	8	6
Mitotic arrest FGCs			17	18	5	7
Soma	5	8	9	6	5	6
Sum	19	29	43	43	18	19
Total	171					

b

Gender	Female embryos	
Week	10W	17W
Mitotic FGCs	34	21
Meiotic FGCs		41
Oogenesis FGCs		12
Soma	7	16
Sum	41	90
Total	131	

c

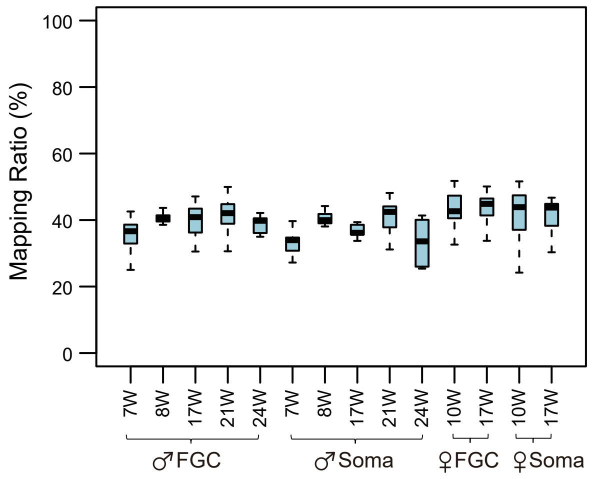

d

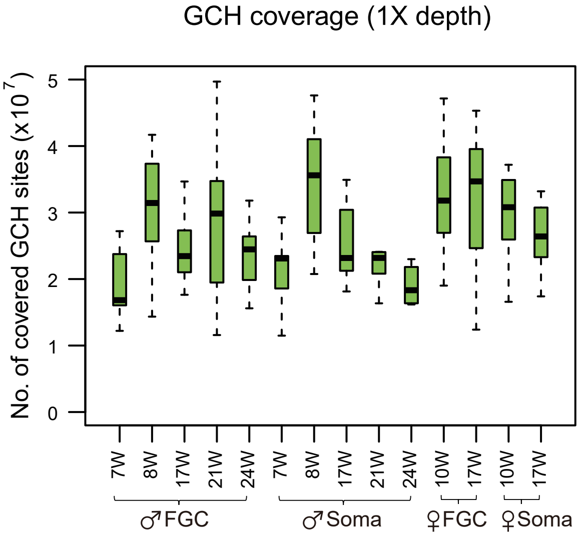

e

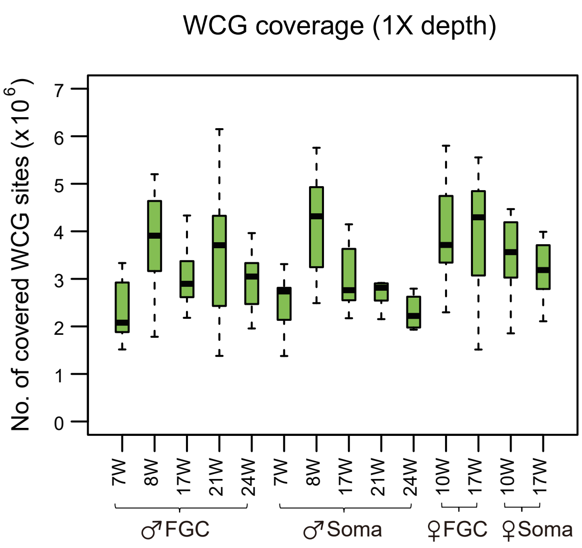

f

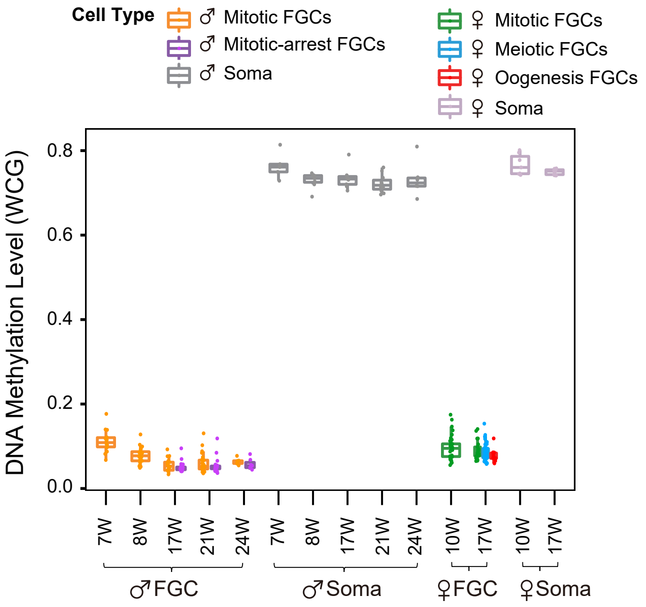

Fig. S4: The sequencing statistics and DNA methylation levels deduced from the scCOOL-seq data.

a and b Overview of the number of male **(a)** and female **(b)** FGCs at different phases and somatic cells analyzed using scCOOL-seq, *in vitro* culture samples were not included.

c Boxplot showing the read mapping ratio of the scCOOL-seq data.

d and e Boxplots of the number of GCH sites **d** and WCG sites **e** covered in a single cell during development at a 1× sequencing depth.

f The DNA methylation level estimated based on a 1× sequencing depth of WCG methylation level in the scCOOL-seq data.
