# Supplementary material for: Dissecting the epigenomic dynamics of human fetal germ cell development at single-cell resolution
Source: Cell Res. 2020 Sep 3;31(4):463–77. doi: 10.1038/s41422-020-00401-9 (PMC8115345; doi:10.1038/s41422-020-00401-9)
Supplement: Supplementary file 5 — Supplementary information, Fig. S5 [file 41422_2020_401_MOESM5_ESM.pdf]

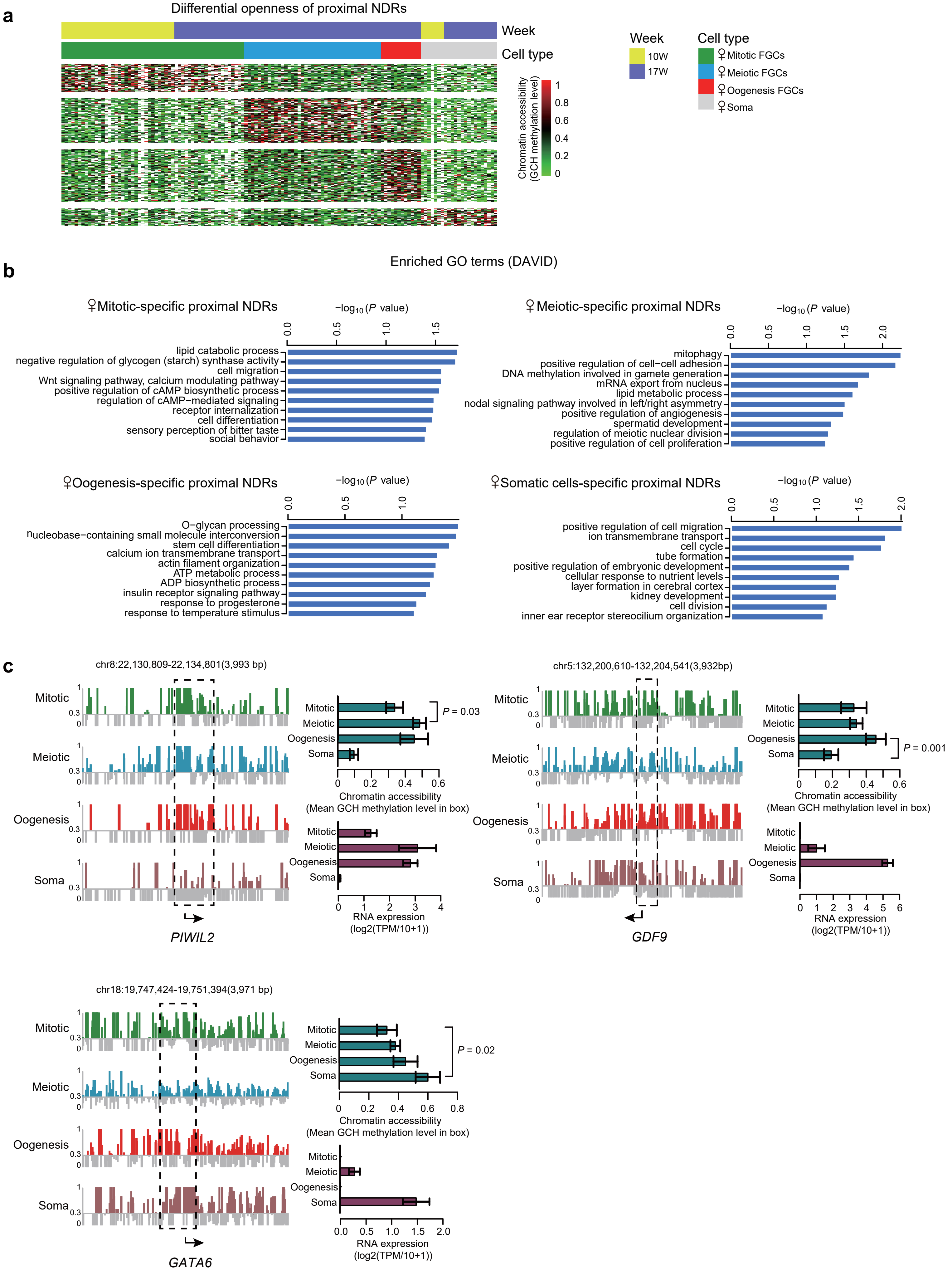
a
Diifferential openness of proximal NDRs
Week
Cell type
Chromatin accessibility (GCH methylation level)
1
0.8
0.6
0.4
0.2
0
Week
10W
17W
Cell type
♀Mitotic FGCs
♀Meiotic FGCs
♀Oogenesis FGCs
♀Soma
b
Enriched GO terms (DAVID)
♀Mitotic-specific proximal NDRs
−log10 (P value)
lipid catabolic process
negative regulation of glycogen (starch) synthase activity
cell migration
Wnt signaling pathway, calcium modulating pathway
positive regulation of cAMP biosynthetic process
regulation of cAMP−mediated signaling
receptor internalization
cell differentiation
sensory perception of bitter taste
social behavior
♀Meiotic-specific proximal NDRs
mitophagy
positive regulation of cell−cell adhesion
DNA methylation involved in gamete generation
mRNA export from nucleus
lipid metabolic process
nodal signaling pathway involved in left/right asymmetry
positive regulation of angiogenesis
spermatid development
regulation of meiotic nuclear division
positive regulation of cell proliferation
♀Oogenesis-specific proximal NDRs
O−glycan processing
nucleobase−containing small molecule interconversion
stem cell differentiation
calcium ion transmembrane transport
actin filament organization
ATP metabolic process
ADP biosynthetic process
insulin receptor signaling pathway
response to progesterone
response to temperature stimulus
♀Somatic cells-specific proximal NDRs
positive regulation of cell migration
ion transmembrane transport
cell cycle
tube formation
positive regulation of embryonic development
cellular response to nutrient levels
layer formation in cerebral cortex
kidney development
cell division
inner ear receptor stereocilium organization
c
chr8:22,130,809-22,134,801(3,993 bp)
Mitotic
Meiotic
Oogenesis
Soma
PIWIL2
P = 0.03
Chromatin accessibility (Mean GCH methylation level in box)
RNA expression (log2(TPM/10+1))
chr5:132,200,610-132,204,541(3,932bp)
GDF9
P = 0.001
chr18:19,747,424-19,751,394(3,971 bp)
GATA6
P = 0.02

Fig. S5: The differential openness of proximal NDRs in female FGCs and gonadal somatic cells.

a Heatmap showing 857 differentially open proximal NDRs among female 10- and 17-week FGCs at sequential phases and somatic cells during development.

b GO analysis of the corresponding genes associated with differentially open proximal NDRs in each cell types.

c The chromatin accessibility around the TSSs of *PIWIL2*, *GDF9* and *GATA6* in the female 17-week FGCs and the somatic cells are shown at a single-base resolution. The cytosines with their methylation level less than 0.3 were shown in gray, and only the cytosines with their methylation level greater than 0.3 were shown in green /blue /red /brown based on the cell types. The representative differentially open regions between cell types are highlight in dashed rectangles. The mean chromatin accessibility of representative regions and RNA expression levels of genes (estimated from female 18-week embryos in our previous study) are shown in the right panel[11]. The direction of the black arrow under each panel represents the direction of transcription. The bar plot and histogram represent means, while the error bars represent s.e.m. values. Statistical significance analysis for the differences of GCH levels between two cell types were performed and the *P* values with Student's t-test were shown here.
