# Supplementary material for: Dissecting the epigenomic dynamics of human fetal germ cell development at single-cell resolution
Source: Cell Res. 2020 Sep 3;31(4):463–77. doi: 10.1038/s41422-020-00401-9 (PMC8115345; doi:10.1038/s41422-020-00401-9)
Supplement: Supplementary file 6 — Supplementary information, Fig. S6 [file 41422_2020_401_MOESM6_ESM.pdf]

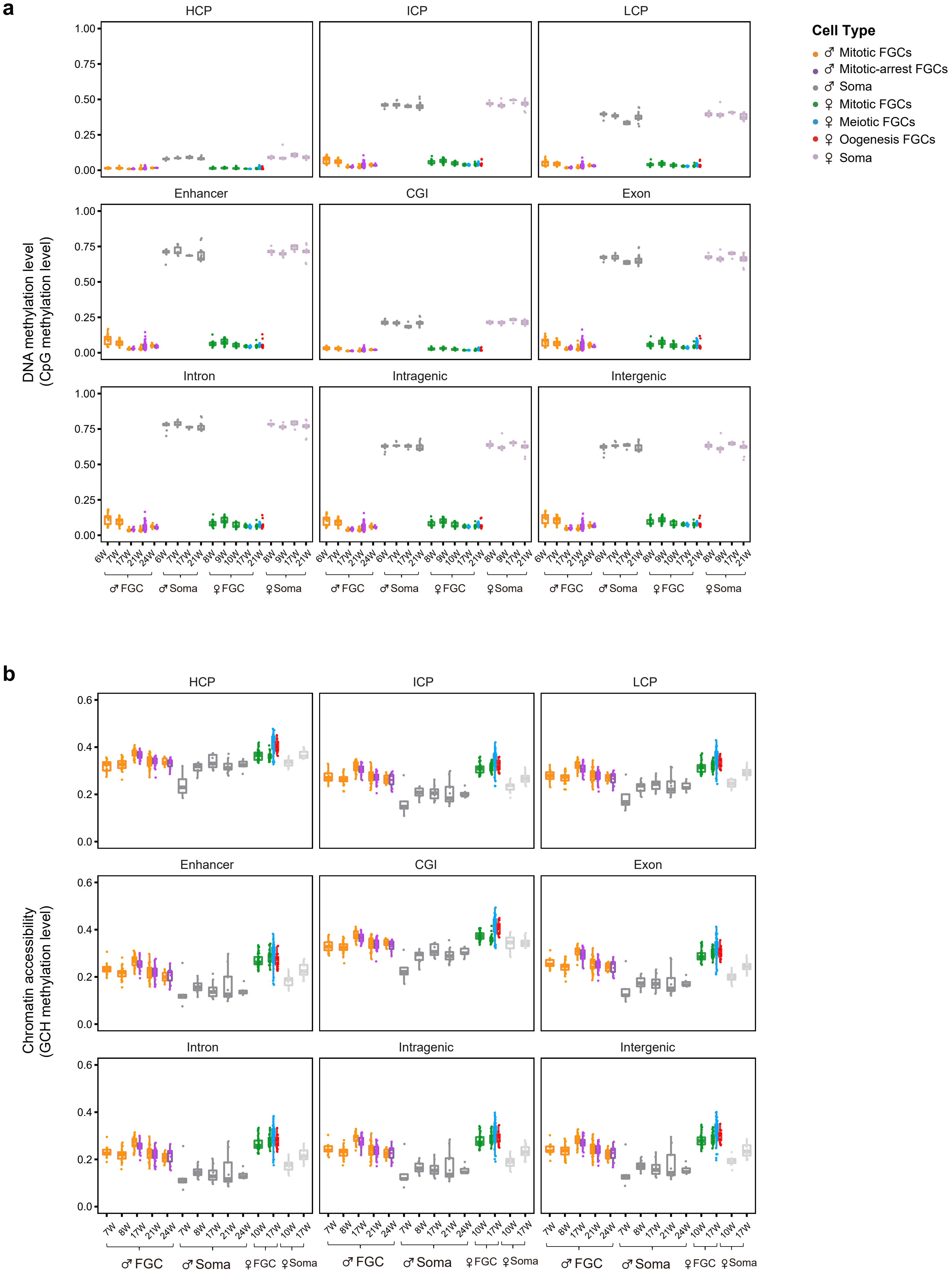
a
HCP
ICP
LCP
Enhancer
CGI
Exon
Intron
Intragenic
Intergenic
DNA methylation level
(CpG methylation level)
1.00
0.75
0.50
0.25
0.00
6W 7W 17W 21W 24W 6W 7W 17W 21W 8W 9W 10W 17W 21W 8W 9W 17W 21W
♂FGC ♂Soma ♀FGC ♀Soma
Cell Type
♂ Mitotic FGCs
♂ Mitotic-arrest FGCs
♂ Soma
♀ Mitotic FGCs
♀ Meiotic FGCs
♀ Oogenesis FGCs
♀ Soma
b
Chromatin accessibility
(GCH methylation level)
0.6
0.4
0.2
0.0
7W 8W 17W 21W 24W 7W 8W 17W 21W 24W 10W 17W 10W 17W
♂ FGC ♂ Soma ♀FGC ♀Soma

Fig. S6: DNA methylation levels and chromatin accessibility of different genomic regions.

a Boxplot showing the DNA methylation levels of various genomic regions at different gestational time points using the scBS-seq data.

b Boxplot showing the chromatin accessibility of corresponding regions during development using the scCOOL-seq data.
