# Supplementary material for: Dissecting the epigenomic dynamics of human fetal germ cell development at single-cell resolution
Source: Cell Res. 2020 Sep 3;31(4):463–77. doi: 10.1038/s41422-020-00401-9 (PMC8115345; doi:10.1038/s41422-020-00401-9)
Supplement: Supplementary file 7 — Supplementary information, Fig. S7 [file 41422_2020_401_MOESM7_ESM.pdf]

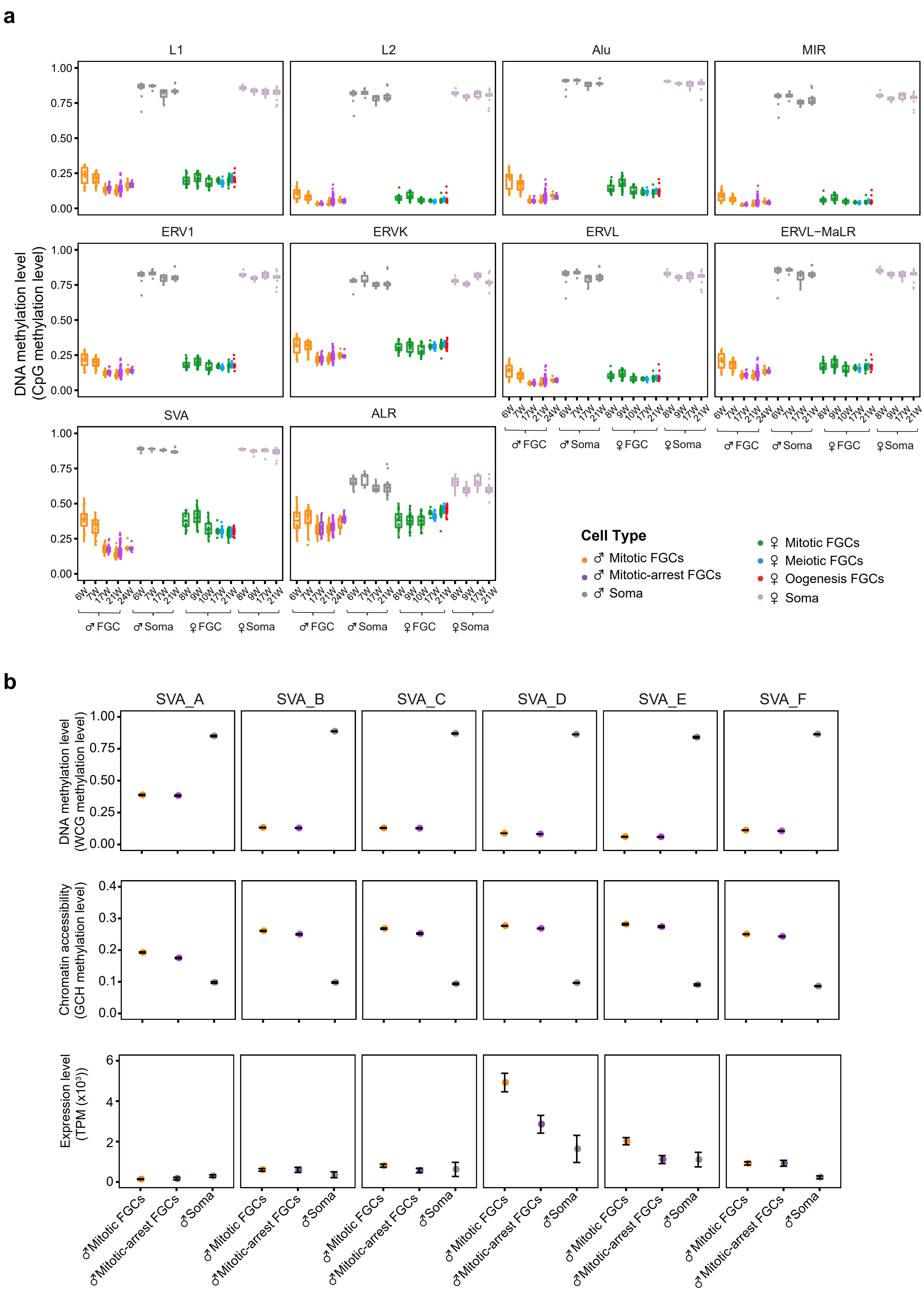
a
L1
L2
Alu
MIR
ERV1
ERVK
ERVL
ERVL−MaLR
SVA
ALR
DNA methylation level
(CpG methylation level)
Cell Type
♂ Mitotic FGCs
♂ Mitotic-arrest FGCs
♂ Soma
♀ Mitotic FGCs
♀ Meiotic FGCs
♀ Oogenesis FGCs
♀ Soma
♂FGC
♂Soma
♀FGC
♀Soma
b
SVA_A
SVA_B
SVA_C
SVA_D
SVA_E
SVA_F
DNA methylation level
(WCG methylation level)
Chromatin accessibility
(GCH methylation level)
Expression level
(TPM (x10³))
♂Mitotic FGCs
♂Mitotic-arrest FGCs
♂Soma

Fig. S7: DNA methylation levels of repetitive elements and features of six SVA subfamilies in male gonads.

a Boxplot showing the DNA methylation levels of various repeats at different gestational time points using the scBS-seq data.

b Average DNA methylation levels, chromatin accessibility and expression levels of six SVA subfamilies in male 21-week embryos. The expression levels were estimated from the RNA-seq data for male 21-week embryos from our previous study[7].
